# Populations of Stored Product Mite *Tyrophagus putrescentiae* Differ in Their Bacterial Communities

**DOI:** 10.3389/fmicb.2016.01046

**Published:** 2016-07-12

**Authors:** Tomas Erban, Pavel B. Klimov, Jaroslav Smrz, Thomas W. Phillips, Marta Nesvorna, Jan Kopecky, Jan Hubert

**Affiliations:** ^1^Biologically Active Substances in Crop Protection, Crop Research InstitutePrague, Czech Republic; ^2^Department of Ecology and Evolutionary Biology, University of Michigan, Ann ArborMI, USA; ^3^Faculty of Biology, Tyumen State UniversityTyumen, Russia; ^4^Department of Zoology, Faculty of Science, Charles University in PraguePrague, Czech Republic; ^5^Department of Entomology, Kansas State University, ManhattanKS, USA

**Keywords:** *Tyrophagus putrescentiae*, bacteria, symbiont, feeding, *Blattabacterium*, *Wolbachia*, 16S rRNA

## Abstract

**Background:** *Tyrophagus putrescentiae* colonizes different human-related habitats and feeds on various post-harvest foods. The microbiota acquired by these mites can influence the nutritional plasticity in different populations. We compared the bacterial communities of five populations of *T. putrescentiae* and one mixed population of *T. putrescentiae* and *T. fanetzhangorum* collected from different habitats.

**Material:** The bacterial communities of the six mite populations from different habitats and diets were compared by Sanger sequencing of cloned 16S rRNA obtained from amplification with universal eubacterial primers and using bacterial taxon-specific primers on the samples of adults/juveniles or eggs. Microscopic techniques were used to localize bacteria in food boli and mite bodies. The morphological determination of the mite populations was confirmed by analyses of CO1 and ITS fragment genes.

**Results:** The following symbiotic bacteria were found in compared mite populations: *Wolbachia* (two populations), *Cardinium* (five populations), *Bartonella*-like (five populations), *Blattabacterium*-like symbiont (three populations), and *Solitalea*-like (six populations). From 35 identified OTUs_97_, only *Solitalea* was identified in all populations. The next most frequent and abundant sequences were *Bacillus, Moraxella, Staphylococcus, Kocuria*, and *Microbacterium*. We suggest that some bacterial species may occasionally be ingested with food. The bacteriocytes were observed in some individuals in all mite populations. Bacteria were not visualized in food boli by staining, but bacteria were found by histological means in ovaria of *Wolbachia*-infested populations.

**Conclusion:** The presence of *Blattabacterium-*like, *Cardinium, Wolbachia*, and *Solitalea*-like in the eggs of *T. putrescentiae* indicates mother to offspring (vertical) transmission. Results of this study indicate that diet and habitats influence not only the ingested bacteria but also the symbiotic bacteria of *T. putrescentiae*.

## Introduction

Mites, like insects, derive nutritive advantages from persistent associations with microorganisms (Van Asselt, 1999; Dillon and Dillon, 2004; Douglas, 2015). Microorganisms associated with insects can synthetize various nutrients, provide essential amino acids and contribute to digestive processes (Dillon and Dillon, 2004; Douglas, 2009). Domestic mites are inhabitants of human-related habitats such as homes, carpets, beds, and stored food (Spieksma, 1997). Although these mites are commensals on the trophic level, due to allergen production and vectoring their microorganisms are of medical importance (Colloff, 2009). It is hypothesized that mites invaded human-related environments through two different routes: house dust mites (HDMs; e.g., *Dermatophagoides pteronyssinus* and *D. farinae*) likely shifted from a parasitic lifestyle back to a commensal life style (Klimov and OConnor, 2013), while ancestors of stored product mites (SPMs; e.g., *Acarus siro, Lepidoglyphus destructor, Tyrophagus putrescentiae*) were free-living and invaded human houses via the nests of birds and small mammals (OConnor, 1979, 1982).

*Tyrophagus putrescentiae* (Schrank, 1781) has been reported from agricultural soils (Smrz and Jungova, 1989), commercial bumblebee colonies (Rozej et al., 2012), and the nests of birds and small mammals (Solarz et al., 1999). This mite is very common in human-created habitats such as dust in urban environments, medical and laboratory facilities, farms, the food industry (Franz et al., 1997; Solarz et al., 2007), and in fungal and insect cultures in laboratories (Duek et al., 2001). The most typical food sources of *T. putrescentiae* are protein and fat-rich substances such as grain germ, nuts, sunflower, oil rape seeds, cheese, ham, and dry dog food (Zakhvatkin, 1959; Robertson, 1961; Hughes, 1976; Garcia, 2004; Palyvos et al., 2008; Erban et al., 2015, 2016; Rybanska et al., 2015). Our broad hypothesis in work reported here is that successful colonization of such a wide range of habitats is facilitated by an inherently broad food plasticity in *T. putrescentiae* that is likely due to symbiotic microbes.

In general, the ancestors of stored-product mites were fungivorous (OConnor, 1979, 1982). Fungivory is well-documented for *T. putrescentiae* (Smrz and Catska, 1987; Hubert et al., 2004; Nesvorna et al., 2012). The mite can nutritionally benefit from interactions with bacteria or both fungi and bacteria. *T. putrescentiae* produces bacteriolytic enzymes which hydrolyze the cell walls of gram positive bacteria (Erban and Hubert, 2008). *T. putrescentiae* has been found to host bacterial communities in the gut, parenchymal tissues and reproductive tract with various interactions (Hubert et al., 2012a; Kopecky et al., 2014a,b). *T. putrescentiae* is associated with bacterial parasites or symbionts (*Cardinium* and *Wolbachia*) inhabiting the reproductive tract and fat body (Kopecky et al., 2013; Brown and Lloyd, 2015). Another association was described for neutral and alkaline proteases, and exo-chitinase producing *Bacillus cereus* in *T. putrescentiae*. *B. cereus* was presented in all samples and the exoenzymes can interact to utilization of the food sources for *T. putrescentiae*; however, the addition of *B. cereus* to the diet led to a substantial suppression of mite population growth (Erban et al., 2016). In contrast, another bacterium, *Micrococcus lysodeikticus*, had no significant influence on the population growth of *T. putrescentiae* (Erban and Hubert, 2008). However, those associated bacteria affect the fitness of mites; therefore, their indirect effects on habitat colonization or diet utilization by mites are expected.

The above facts suggest that interactions of mites with microorganisms are important for adaptation to a nutritional food source in human-made habitats as well as in soil. A diet switches from plant-derived food to various fungal species caused changes in the bacterial community associated with the gut and parenchymal tissues of *T. putrescentiae* (Smrz, 2003; Hubert et al., 2012b) and induced bacteriocytes (sometimes called as extraintestinal bacterial bodies or bacteriome; Smrz and Catska, 1989; Smrz, 2003; Smrz and Soukalova, 2008). It is likely that *T. putrescentiae* acquires bacteria with chitinolytic activity (i.e., *B. cereus* and *Serratia marcescens*) to digest chitin from fungal cell walls or mite bodies (Smrz et al., 1991; Smrz and Catska, 2010; Erban et al., 2016). The bacteria producing exo-chitinases can contribute to degradation of mite exuviae, mite bodies or food boluses consisting of chitin, including the peritrophic membrane (Erban et al., 2016).

In laboratory experiments adding antibiotics to the diet did not eliminate the bacteria in *T. putrescentiae*; *Kocuria* and *Bacillus* were still present in surface cleaned mite body homogenates (Kopecky et al., 2014b). Our recent study showed a population-specific density-dependent growth of *T. putrescentiae* (Rybanska et al., 2015) and also indicated the possibility that *T. putrescentiae* populations may differ in acquired bacteria (Smrz and Catska, 2010), suggesting a possible habitat influence on mite internal bacteria.

Here, we compare bacterial community of six distinct populations of *T. putrescentiae*. The comparison is based on Sanger sequencing of bacterial 16S rRNA genes from adults/juveniles. Because we found diverse symbiotic bacterial community in these populations, we also focused on identification of these symbiotic bacteria in the eggs by bacterial taxa specific primers. Finally, we compared sex ratios and guanine contents in *T. putrescentiae* populations in relation to the presence of symbiotic bacteria. Limitations associated with the small amount of starting wild material do not allow reliable intra-population comparison of bacterial communities because prolonged mite culture maintenance under standardized laboratory conditions can change bacterial communities.

## Materials and Methods

### Mites

The bacterial communities of three field and three laboratory populations of *Tyrophagus putrescentiae* (Schrank, 1781) were compared (**Table 1**). Morphological determination of populations was performed by under a compound microscope using characters described in Klimov and OConnor (2009a,b). In addition, molecular markers were used for subsequent characterization of the populations. All these *T. putrescentiae* populations were placed into IWAKI 25 cm^2^ surface area 70 mL tissue-culture flasks (IWAKI flasks; Cat. No. 3100-025; Sterilin, Newport, UK) with their original food (see **Table 1**), separately. The flasks were placed into Secador desiccator cabinets (Bel-Art Products, Pequannock, NJ, USA) and incubated under controlled conditions at 25 ± 1°C and 85% RH in darkness for up to 3 months; then the mites were sampled.

**Table 1:** Sampled populations of *Tyrophagus putrescentiae* and their habitats at the time of collection.

Abbreviations	Population	Habitat	Food	Collected		
				Year	Collector	Site
Ham	Ham	Field	Ham	2013	A. Sala	Cesena, Italy
Kop	Koppert	Laboratory	Grain-derived	2012	E. Baal	The Netherlands
Lab	Laboratory	Laboratory	Grain-derived	1996	E. Zdarkova	Bustehrad, Czech Republic
Dog	Dog	Field	Dry dog food	2007	J. Hubert	USA
Phi	Phillips	Laboratory	Dry dog food	2014	T. Phillips	USA
Zvo	Zvoleneves	Field	Grain-derived	2011	M. Nesvorna	Zvoleneves, Czech Republic

*The year indicates since the colony was maintained in the Crop Research Institute, Prague, Czech Republic.*

*Tyrophagus putrescentiae* mites from field populations were reared for up to 1–2 months in the laboratory. During this time the mite populations multiplied to a level at which sampling of many individuals was possible. For DNA extraction, mites were collected and separated from food particles using a fine-tipped artist’s paint brush, transferred into Eppendorf tubes, and weighed using a microbalance (MS Mettler-Toledo, Greifensee, Switzerland) to obtain 0.05 ± 0.01 g wet weight samples in triplicates per populations. The mean of fresh weight of *T. putrescentiae* individuals is about 8 μg, so every sample contains ca 6,300 individuals. The Eppendorf tubes with collected mites were filled with 80% ethanol and stored in a refrigerator at 4°C until DNA extraction. Each population of *T. putrescentiae* was processed in triplicate. For the comparison of the bacterial community of the laboratory population (**Table 1**) we used previously published sequences of bacteria from laboratory strains (Hubert et al., 2012a,b; Kopecky et al., 2014b). For microanatomical samples, the mites of approximately 100–500 individuals were fixed in modified Bouin-Dubosque-Brasil fluid according to Smrz (1989).

We adopted a method of Stepien and Rodriguez (1973) for eggs accumulation and extraction. The food with mites from rearing flasks was placed on mesh with a size of 176 μm under the water surface. All of the mesh used was polyamide fiber (Silk & Progress, Brnenec, Czech Republic). The females deposited eggs on the water surface after 48 h (Hubert et al., under review). The water was collected and filtrated though the mesh manifold (Stepien and Rodriguez, 1973) using a vacuum pump. The mesh sizes in the manifold were in decreasing order: 411, 300, 206, 176, 139, 109, 86, 42 μm diameter. Next, the eggs were cleaned with ddH_2_0, Tween^®^ 20 (Cat No. P9416, Sigma-Aldrich), bleach, and 80% ethanol (Hubert et al., under review). The eggs were captured at 86 and 42 μm mesh and removed by pipetting into Eppendorf tubes and stored in 80% ethanol. Each sample consisted of 50 eggs in triplicate per population.

### Sample Homogenization and DNA Extraction

Prior to DNA extraction, ethanol was replaced, in sequence, by bleach, and then washed three times with sterile phosphate-buffered saline (PBST: 3.2 mM Na_2_HPO_4_, 0.5 mM KH_2_PO_4_, 1.3 mM KCl, and 135 mM NaCl) with 0.05% w/w Tween^®^ 20 detergent (Cat No. P9416, Sigma-Aldrich, St. Louis, MO, USA) to remove surface microflora. Samples with a total volume of 100 μL PBST were homogenized in a Radnoti tissue grinder (Cat. No. 440613; Monrovia, CA, USA). DNA from the homogenates was extracted using a Wizard^®^ SV Genomic DNA Purification System (Cat No. A2361, Promega) according to the manufacturer’s instructions. Extracted DNA was stored in a freezer at -20°C until the analyses were performed.

### Molecular Markers for Identification of *T. putrescentiae* Populations

The primers (**Table 2**) spanning a portion of the 5.8S ribosomal DNA (rDNA), the full-length ITS2 region, and a part of the 28S rRNA and primers for the central part of the CO1 region of *T. putrescentiae* were used to confirm species determinations for mites used in this research (Yang et al., 2011). Amplifications were performed in a C1000 Thermal Cycler (Bio-Rad, Hercules, CA, USA). A total volume of 25 μl polymerase chain reaction (PCR) reaction mixture contained 200 μM dNTPs, 3 mM MgCl_2_, forward and reverse primers (100 nM each), 0.5 unit Taq polymerase (all from Promega), and 5–30 ng of template DNA that included mite genomic DNA (for reaction conditions, see Yang et al., 2011). The amplicons were done for every sample. The resulting PCR products were visualized by agarose gel electrophoresis. Because we used samples at the population level, amplicons were purified with Wizard^®^ SV Gel and the PCR product clean-up system Kit (Cat No. A9281, Promega) and cloned using pGEM^®^-T Easy Vector (Cat No. A1380 Promega). We usually selected four clones per sample, i.e., 12 clones per population. Selected clones were sequenced by Macrogen (Seoul, South Korea). The obtained sequences were assembled with CodonCode Aligner, version 5.1.5 (CodonCode Corporation, Dedham, MA, USA).

**Table 2:** Primers used for characterization of *Tyrophagus putrescentiae* populations and detection of bacterial community.

Specificity	Target	Name	Primer 5′–3′	Tm (°C)	Length (bp)	Reference
*T. putrescentiae* -genomic	ITS2 region	28S-F	CGACTTTCGAACGCATATTGC	55	488	Yang et al., 2011
		28S-R	GCTTAAATTCAGGGGGTAATCTCG			
	CO1 region	CO1-F	GTTTTGGGATATCTCTCATAC	50	377	Yang et al., 2011
		CO1-R	GAGCAACAACATAATAAGTATC			
*Bacteria*	16S rRNA	F27	AGAGTTTGATCCTGGCTCAG	50	1460	Lane, 1991
		R1492	TACGGYTACCTTGTTACGACTT			
*Wolbachia*	16S rRNA	WpF	TTGTAGCCTGCTATGGTATAACT	52	900	O’Neill et al., 1992
		WpR	GAATAGGTATGATTTTCATGT			
*Bartonella*-like	16S rRNA	Bart 1F	TGTCWCCGAYCCAGCCK	63	920	Kopecky et al., 2014a
		Bart 2R	TGTCTCCGACCCAGCCT			
*Cardinium*	16S rRNA	Card4	CTTAACGCTAGAACTGCGA	55	800	Kopecky et al., 2013
		Card6	TCAAGCTCTACCAACTCC			
	16S rRNA	Card1F	CGCATGCAATCTACTTTACAC	55	1314	This study
		Card1R	GCCACTGTCTTCAAGCTCTAC			
*Blattabacterium*	16S rRNA	35F	TGCAAGTCGAGGGGC	62	1260	Clark and Kambhampati, 2003
		1294R	GTCGAGTTGCAGACTCCAATC			
*Solitalea*-like	16S rRNA	Soli F	TGCGACACAAAGAGCTGA	54	670	This study
		Soli R	GCTGGCAACAGTACATGG			
*Spiroplasma*	16S rRNA	BS1	AAGTCGAACGGGGTGCTT	57	975	Meeus et al., 2012
		BS976	TGCACCACCTGTCTCAATGT			

### Description of the Bacterial Community

Polymerase chain reaction amplification of 16S rRNA gene fragments was used to characterize the bacterial community using universal 27F/1492R primers (Barbieri et al., 2001). The PCR was done for every sample with the exception of laboratory population of *T. putrescentiae*. For the latter population, PCR conditions were same as was described above with the exception of annealing temperature (**Table 2**). The PCR products were cloned using the same protocol as above and 12 colonies were usually selected per sample (i.e., 30 per population) and sequenced in Macrogen. The same laboratory population of *T. putrescentiae* was analyzed by the same design on the same diet in our previous studies (Hubert et al., 2012a,b; Kopecky et al., 2014b) and we used the clones to characterize the bacterial community of *T. putrescentiae* laboratory population.

The presence of *Cardinium, Wolbachia, Bartonella*-like bacteria, *Solitalea*-like bacteria and *Blattabacterium*-like bacteria was determined by using taxon-specific primers (see **Table 2**). As a positive control, we used DNA samples of mites where bacteria had previously been identified using the same methodology (Kopecky et al., 2013, 2014a). The negative control was the double-distilled water used for the PCR master mix preparation. The reaction conditions were the same as described for the molecular markers used for identification of *T. putrescentiae* populations; the amplification conditions are specified in **Table 2**. All samples were tested in triplicate. PCR product of the expected size was considered as positive sample. One positive sample was considered as positive presence of bacteria in the population. A negative sample was a sample with no product detected by specific primers. The primer specificity was checked by sequencing of the randomly selected amplicons according to protocol described above.

### 16S rRNA Sequences

The 16S rRNA sequences were obtained by cloning of amplicons of universal eubacterial primers 27F/1492R (**Table 2**). The obtained sequences were assembled with CodonCode Aligner and the chimeras were removed using the Mallard and Pintail software (Ashelford et al., 2005, 2006). Altogether, 262 sequences obtained in this study and 176 sequences from previous studies (Hubert et al., 2012a,b; Kopecky et al., 2014b). The sequences were analyzed in MOTHUR v.1.36.0 software (Schloss et al., 2009). The sequences were aligned to Silva reference database (Quast et al., 2013), filtered and clustered and then analyzed and assigned to the operational taxonomic units (OTUs) defined at a distance level of 0.03. The sequences were identified using the Ribosomal Database Project’s (RDP) naive Bayesian rRNA classifier as training set No. 14 (Wang et al., 2007). The representative sequences for individual OTUs_97_ were compared to the sequences in GenBank using nucleotide Blastn (Altschul et al., 1997).

### Phylogenetic Analysis

We conducted phylogenetic analyses on sequences of 16S rRNA, ITS-2 and CO1 of Flavobacteriales symbionts and *Wolbachia*. The references sequences originated from GenBank or RDP. Alignments of partial 16S rRNA gene sequences were performed using the SILVA Incremental Aligner v.1.2.11 (Pruesse et al., 2012). For the analysis of phylogenetic relationships, the best-fit model of nucleotide substitution was selected using jModelTest v.2.1.7 (Guindon and Gascuel, 2003; Darriba et al., 2012). Based on the selection, the general time reversible model with a proportion of invariable sites and gamma distribution (GTR+G+I), was employed to infer phylogenies in a Bayesian framework in PhyloBayes-MPI, v.1.4e (Lartillot et al., 2009) and the maximum likelihood framework in PhyML v.3.0 (Guindon et al., 2010). Phylograms were visualized in MEGA 6 (Tamura et al., 2007).

### Microanatomical Analyses

#### Sections

Mites were fixed in modified Bouin-Dubosque fluid (Smrz, 1989) for 3 days and then transferred to paraffin. The fixation fluid was replaced by 100% isopropyl alcohol for 12 h (two times), isopropyl alcohol/methyl benzoate (1/1 v/v) for 12 h (two times), methyl benzoate for 12 h (two times), benzene for 2 h, benzene/paraffin (1/1 v/v) for 12 h at 48°C, and paraffin for 12 h (two times) at 56°C (Hubert et al., 1999). Mites were transferred from containers to Peel-A-Way^®^ embedding molds (Polysciences, Eppelheim, Germany) and embedded in Paraplast Plus^®^ (Cat No. 39602004, Leica Biosystems, Nussloch, Germany) at 56°C. Paraffin blocks were sectioned to 4–6 μm sections on Microm HM 200 ErgoStar Microtome (Carl Zeiss, Jena, Germany).

#### Staining

The slides with sections were placed into different staining solutions equally. The sections were stained by Masson’s triple stain combined with PAS (periodic acid and Schiff agent) and Mann-Dominic and Ziehl–Neelsen staining for bacterial visualization.

#### Observations

At least 15 specimens per population were observed using an Axioskop compound microscope equipped with a digital camera and Axiovision software (Carl Zeiss).

#### Visualization in Microscopic Slides

The mites were mounted on permanent slides in Liquide de-Swan medium (distilled water, 20 mL; gum arabic, 15 g; chloral hydrate 50 g and glucose 3 g, glacial acetic acid 5 mL; Kramar, 1953). The sexing of adults and a quantitative estimate of guanine granulae were performed using a compound microscope. Due to the transparency of the mite body, the granulae were visible without staining (**Figure 9**). At least 100 specimens per one-sex determination and 30 per guanine quantification were observed. The semiquantitative categories were as follows: (0) no guanine granules, (1) low number, the granules filled less than 25% of the hysterosoma, (2) intermediate – granules filled between 25 and 50% of the hysterosoma, and (3) massive, granules filled more than 50% of the hysterosoma (**Figure 9**).

### Statistical Analyses

The similarity of bacterial community in populations was based on analyses of 16S rRNA clones’ library. The shared file was constructed in MOTHUR. The diversity indexes, rarefaction and the comparison of population using Principal coordinate analyses with Euclidian and Jaccard distances were calculated in PAST 3 software (Hammer et al., 2001). The heatmap was constructed in XLSTAT (Addinsoft, New York, NY, USA) using filtering by standard deviation and reduction of low abundant OTUs. The data describing sex ratio and guanine contents of mite bodies were tested by a chi-square test with Bonferroni correction in STATISTIX 9 software (Analytical Software, Tallahassee, FL, USA).

## Results

### The Comparison of CO1 and ITS in *T. putrescentiae* Populations

**FIGURE 1:**
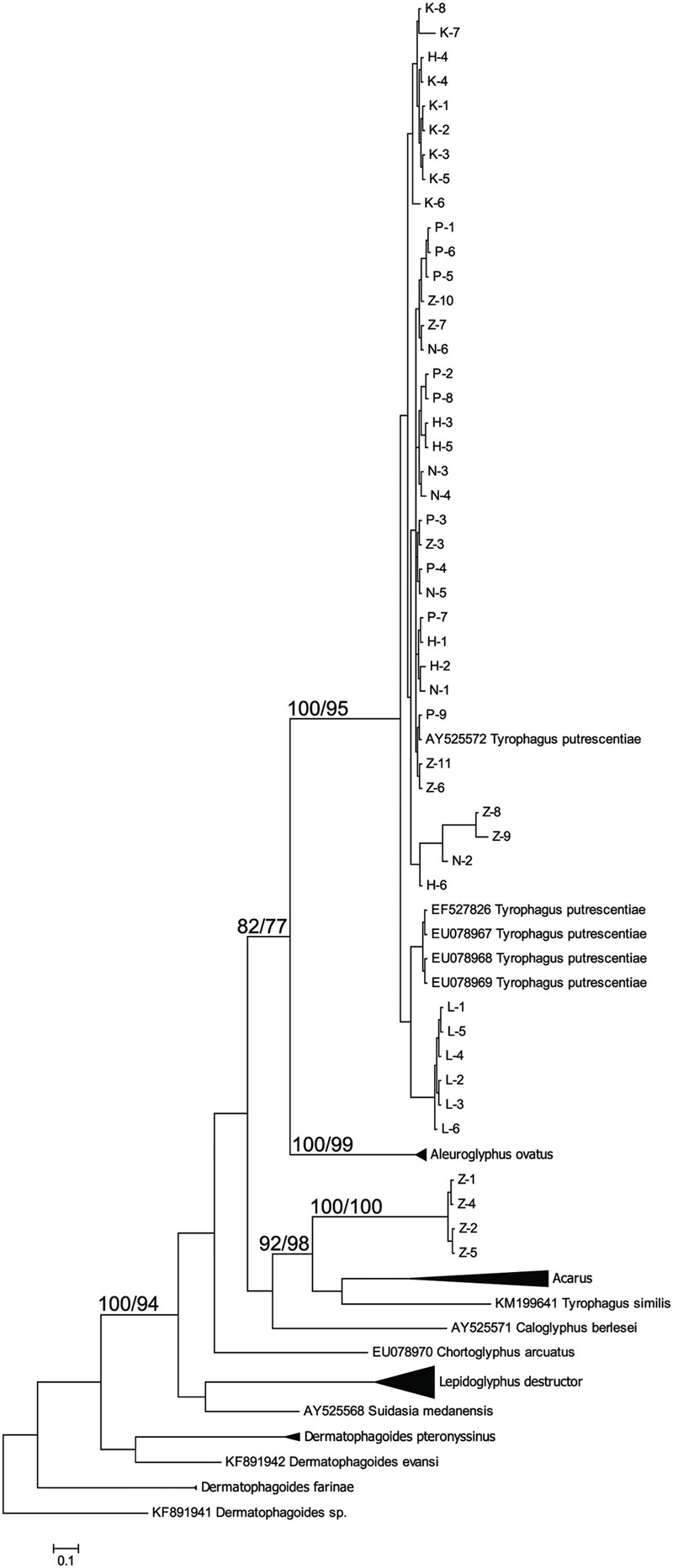
**Phylogenetic analysis of the CO1 clones of *Tyrophagus putrescentiae* with the reference sequences from other related astigmatid mites (Webster et al., 2004; Dermauw et al., 2009; Klimov and OConnor, 2009a,b; Bochkov et al., 2014; Ge et al., 2014; Sun et al., 2014).** The phylogeny was inferred by Bayesian analysis of 46 partial CO1 gene sequences from observed populations, i.e., L, laboratory; K, Koppert; P, Phillips; D, Dog; H, Ham; and Z, field Zvoleneves. Branch lengths correspond to the mean posterior estimates of evolutionary distances (scale bar: 0.5). Branch labels indicate the Bayesian posterior probabilities and bootstrap support values from maximum likelihood analysis.

**FIGURE 2:**
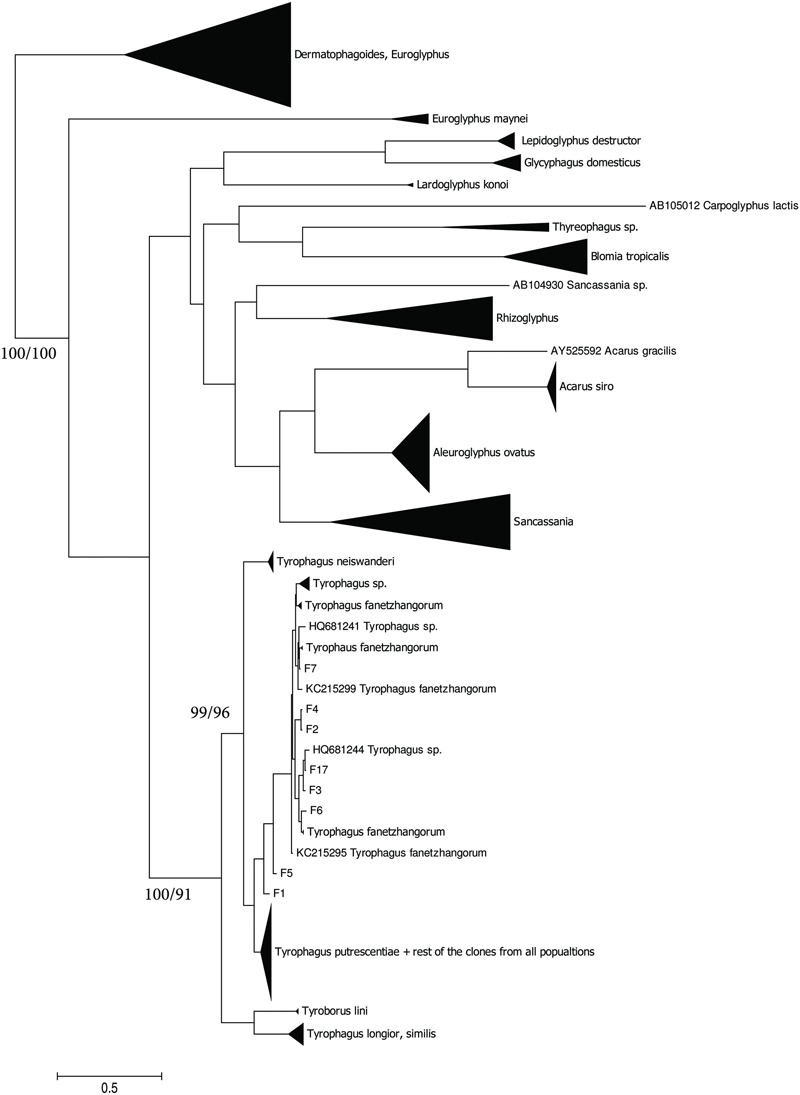
**Phylogenetic analysis of ITS clones of *T. putrescentiae* with the reference sequences from other related astigmatid mites (Noge et al., 2005; Liu et al., 2006; Klimov and OConnor, 2008, 2013; Yang et al., 2011; Beroiz et al., 2014).** The phylogeny was inferred by Bayesian analysis of 46 partial ITS gene sequences from the observed populations, i.e., L, laboratory; K, Koppert; P, Phillips; D, Dog; H, Ham; and Z, field Zvoleneves. Branch lengths correspond to the mean posterior estimates of evolutionary distances (scale bar: 0.1). Branch labels indicate Bayesian posterior probabilities and supporting bootstrap values from maximum likelihood analysis.

The taxonomic comparison based on Bayesian analyses of CO1 showed that observed *T. putrescentiae* populations (i.e., Laboratory, Dog, Koppert, and Phillips) clustered together with the known CO1 sequences from GenBank. The exception occurred with the field populations from Zvoleneves, which formed two separate clusters: (i) four sequences (Z 1, 2, 4, 5) formed one cluster with *A. siro* and (ii) the next sequences clustered to *Tyrophagus similis* (**Figure 1**). Using a diagnostic CO1 dataset of 25 identified species of *Tyrophagus* (PBK, unpublished), we matched these four sequences with *Tyrophagus fanetzhangorum*. Seven other sequences clustered with *T. putrescentiae*, based on both GenBank data and our *Tyrophagus* dataset. The Bayesian analyses of ITS confirm the previous classification for the laboratory populations, Dog, Koppert, and Phillips with *T. putrescentiae* and similar to the CO1 data, the field Zvoleneves population (eight sequences) clustered with *T. fanetzhangorum* (**Figure 2**).

### Flavobacteriales Symbiont (*Blattabacterium*-Like)

**FIGURE 3:**
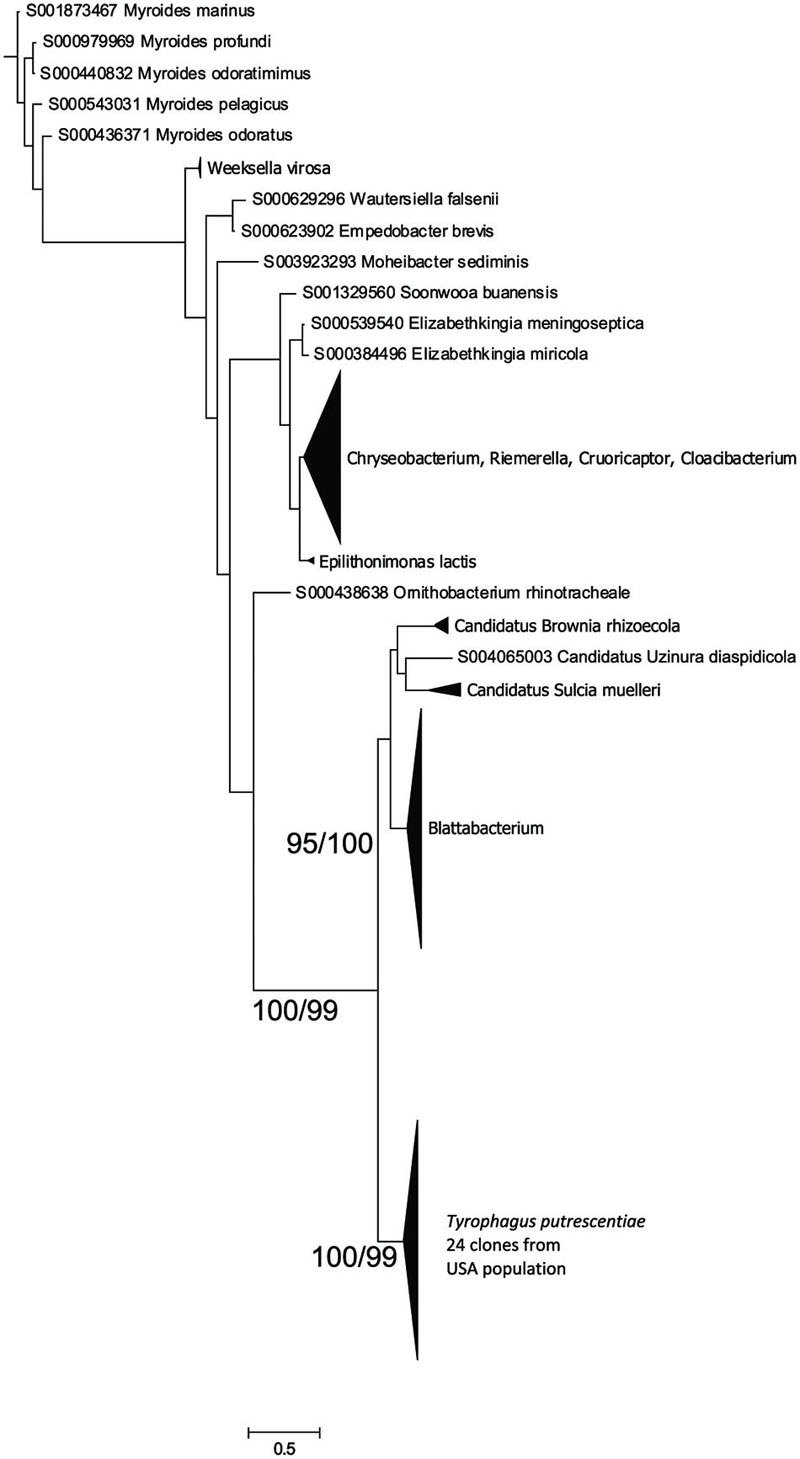
**Phylogenetic analysis of the *Blattabacterium*-like symbiont clones obtained from the Dog population of *T. putrescentiae.*** The phylogeny was inferred by a Bayesian analysis of 24 partial 16S rRNA gene sequences with the reference sequences from RDP, i.e., 126 sequences of *Blattabacterium, C.* Brownia, and *C.* Sulcia, and the sequences of 398 type strains representing the order *Flavobacteriales*. Branch lengths correspond to the mean posterior estimates of evolutionary distances (scale bar: 0.5). Branch labels indicate Bayesian posterior probabilities and bootstrap support values from maximum likelihood analysis. The phylogram was rooted using *Bacteroides fragilis* sequence NR074784 as an outgroup.

The *Blattabacterium* symbiont was amplified using specific primers designed for identification of *Blattabacterium* in cockroaches. The symbiont was present in mite populations from dog food only as indicated by 16S rRNA sequences from eubacterial primers, as well as the amplicons of 16S rRNA fragments obtained by specific *Blattabacterium* primers. A Bayesian analysis of the obtained sequences showed that the sequences clustered as a monophyletic lineage outside *Blattabacterium* (Clark and Kambhampati, 2003), *Candidatus* Brownia rhizoecola (Gruwell et al., 2010), *C.* Uzinora diaspidicola (Gruwell et al., 2007), and *C.* Sulcia muelleri (Moran et al., 2005; **Figure 3**).

### Wolbachia

**FIGURE 4:**
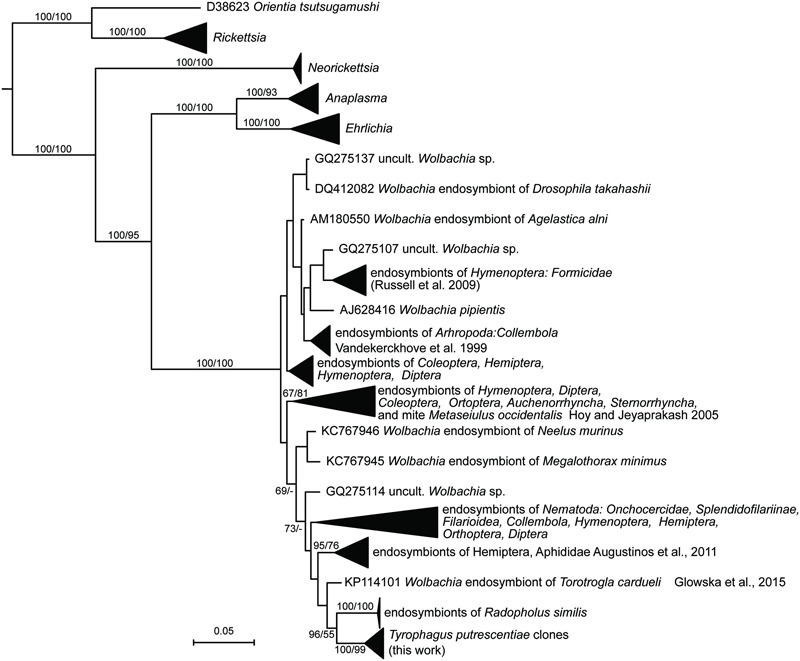
**Phylogenetic analysis of the *Wolbachia* clones obtained from the Dog and Phillips populations of *T. putrescentiae*.** The phylogeny was inferred by a Bayesian analysis of 19 partial 16S rRNA gene sequences cloned from *T. putrescentiae* with the reference sequences from RDP, 242 known *Wolbachia* endosymbionts (Vandekerckhove et al., 1999; Hoy and Jeyaprakash, 2005; Haegeman et al., 2009; Russell et al., 2009; Augustinos et al., 2011; Glowska et al., 2015) and 27 type strains of the order *Rickettsiales*. Branch lengths correspond to the mean posterior estimates of evolutionary distances (scale bar: 0.05). Branch labels indicate Bayesian posterior probabilities and bootstrap support values for maximum likelihood analysis. The phylogram was rooted using *Rhizobium oryzae* sequence EU056823 as an outgroup.

The sequences of *Wolbachia* were found in the clones of amplicons obtained by the clones form *Wolbachia* specific primers (WpF/WpR in **Table 2**) amplicons, i.e., 9 and 10 from Dog and Phillips populations, respectively. A Bayesian analysis of the obtained sequences and sequences in RDP showed that the *T. putrescentiae* sequences formed separate cluster (**Figure 4**). Our analyses showed more clusters of the sequences. The analyses differentiated the sequences from the insects and nematodes, with a few exceptions. The sequences were closer to clusters of nematodes and aphids. The sister group was formed by *Wolbachia* sequences from nematode *Radopholus similis* (Haegeman et al., 2009) and mite *Torotrogla cardueli* (Prostigmata: Syringophilidae; Glowska et al., 2015).

### Comparison of Bacterial Community Based on 16S rRNA Gene

**FIGURE 5:**
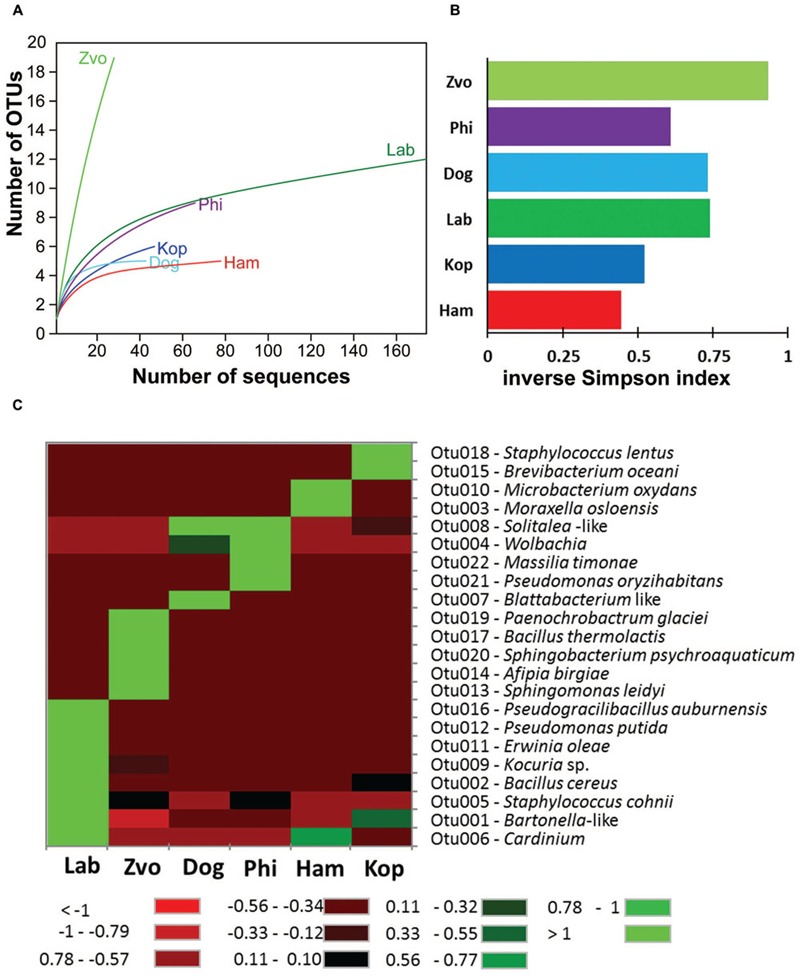
**The diversity and composition of bacterial communities in *T. putrescentiae* populations based on Sanger sequencing of the 16S rRNA gene clones, the amplicons originated from eubacterial primers (F24/R1492): **(A)** rarefaction analyses, **(B)** inverse Simpson diversity index, **(C)** heat map.** Abbreviations for the *T. putrescentiae* populations are listed in **Table 1**.

Obtained 16S rRNA sequences were deposited in GenBank (Accession Numbers: KX022128–KX022390) and combined to 176 sequences of the laboratory *T. putrescentiae* population which are available in GenBank (Accession Numbers: JN236405–JN236431; JX001234–JX001344, KJ635082–KJ635148; Hubert et al., 2012a,b; Kopecky et al., 2014b). All the sequences originated from amplification and cloning the amplicons from universal eubacterial primers. Altogether 42 OTUs_97_ were distinguished (Supplementary Table S1). The diversity of bacterial community was similar for all populations of *T. putrescentiae* with exception of Zvoleneves population (**Figures 5A,B**). The bacterial community of mite population was formed from known/suspected symbionts or parasites: *Bartonella*-like bacteria (OTU_97_ 1), *Wolbachia* (OTU_97_ 4), *Cardinium* (OTU_97_ 6), *Blattabacterium*-like symbiont (OTU_97_ 7), *Solitalea*-like bacteria (OTU_97_ 8; **Figure 5C**). The next most frequent and abundant OTUs_97_ were *Bacillus* (OTU_97_ 2), *Moraxella* (OTU_97_ 3), *Staphylococcus* (OTU_97_ 5), *Kocuria* (OTU_97_ 9), and *Microbacterium* (OTU_97_ 10; Supplementary Table S1).

**FIGURE 6:**
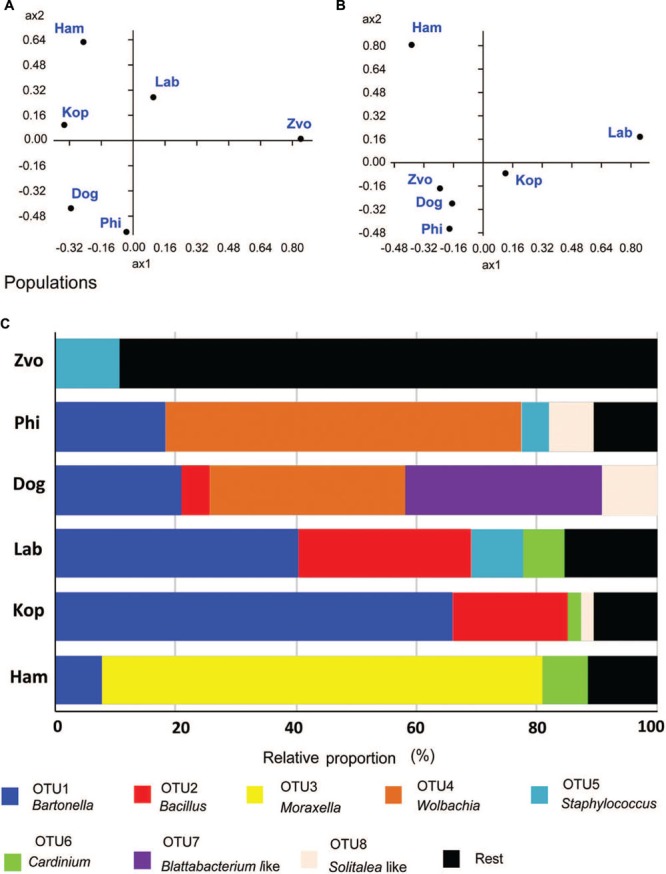
**Comparison of bacterial communities in *T. putrescentiae* populations based on Sanger sequencing of the 16S rRNA gene clones, the amplicons originated from eubacterial primers (F24/R1492): **(A,B)** principal coordinate analyses of bacterial communities in examined mite populations; **(A)** based on Euclidian distance; **(B)** based on Jacquard similarity index; **(C)** relative proportions of cloned bacterial sequences in our 16S rRNA library from different populations of *T. putrescentiae*.** Abbreviations for the *T. putrescentiae* populations are listed in **Table 1**.

The populations differed in observed bacterial community as indicated by the 16S rRNA library. Principal coordinate analyses using Euclidian data matrix showed that bacterial communities of Phillips, and Dog mite populations were similar, while Zvoleneves, Laboratory and Ham populations were formed from different bacteria (**Figure 6A**). The first principal axis explained 60% and the second axis explained 23% of variation in the dataset. When Jaccard data matrix was calculated, the bacterial community was similar in Zvoleneves, Dog and Phillips populations, while Laboratory and Ham populations were different (**Figure 6B**). The first axis explained 59% and second axis explained 29% of variation in the data set. Composition between populations is reported in **Figure 6C**.

To confirm the distribution of bacterial taxa obtained from the analyses of cloned sequences from eubacterial (F24/R1492) amplicons, the samples were analyzed by taxa specific primers. The presence/absence of selected taxa of symbiotic/parasitic bacteria was confirmed by specific primers in the samples of adults and in the eggs (**Table 3**).

**Table 3:** The presence or absence of amplicons of bacterial 16S rRNA generated with universal and taxon-specific primers in different populations of *Tyrophagus putrescentiae*.

Taxa	Population											
	Ham		Kop		Lab		Dog		Phi		Zvo	
	A/J	E	A/J	E	A/J	E	A/J	E	A/J	E	A/J	E
*Bacteria*	+	+	+	+	+	+	+	+	+	+	+	+
*Bartonella*-like	+	-	+	-	+	-	+	-	+	+	-	-
*Cardinium*	+	+	+	+	+	+	-	-	+	+	+	-
*Wolbachia*	-	-	-	-	-	-	+	-	+	+	-	-
*Blattabacterium-*like	-	+	-	-	-	+	+	+	-	-	-	-
*Solitalea*-like	+	+	+	+	+	+	+	+	+	-	+	+
*Spiroplasma*	-	-	-	-	-	-	-	-	-	-	-	-

*A/J – adults/juveniles of mites, E – eggs; PCR product presence or absence is denoted by plus (+) or minus (-) sign.*

The highest number of OTUs was found in the Zvoleneves population, while the lowest was in the Phillips population. Generally, no OTU_97_ (i.e., sequences with 3% of dissimilarity) was shared by all the populations. But when taxa specific primers were used the presence of *Solitalea* was confirmed in all populations including the eggs. The exception was Phillips *T. putrescentiae* population, when the *Solitalea* was not detected in the eggs (**Table 3**). *Bartonella* (OTU_97_ 1) had the highest relative numbers in the Koppert and laboratory strains, but *Bartonella* was present in the Phillips, Dog and Ham populations as well. The amplicon from specific primers confirms the *Bartonella* in all populations except field Zvoleneves population. *Bartonella* was not found in the eggs except of the Phillips *T. putrescentiae* population. *Wolbachia* (OTU_97_ 4) had high relative numbers in the Dog and Phillips populations. The specific primers confirm *Wolbachia* in adults/juveniles of Dog and Phillips populations. However, in the eggs, *Wolbachia* was found only in Dog *T. putrescentiae* population (**Table 3**). *Bacillus* (OTU_97_ 2) formed bacterial communities in the Laboratory and Koppert populations. *Moraxella* (OTU_97_ 3) formed bacterial communities in the Ham population only and the *Blattabacterium*-like symbiont (OTU_97_ 7) in the Dog population only. However, the specific primers confirm *Blattabacterium*-like symbiont in both adults/juveniles of *T. putrescentiae* Dog population and in the eggs of laboratory *T. putrescentiae* population (**Table 3**). *Cardinium* (OTU_97_ 6) was found in the clones of 16S rRNA of Ham, Koppert and Laboratory *T. putrescentiae* population. However, the presence was confirmed in all populations by specific primers with exception of Dog population and in the majority of populations was detected in the eggs.

### Microanatomical Description of Associated Bacteria

**FIGURE 7:**
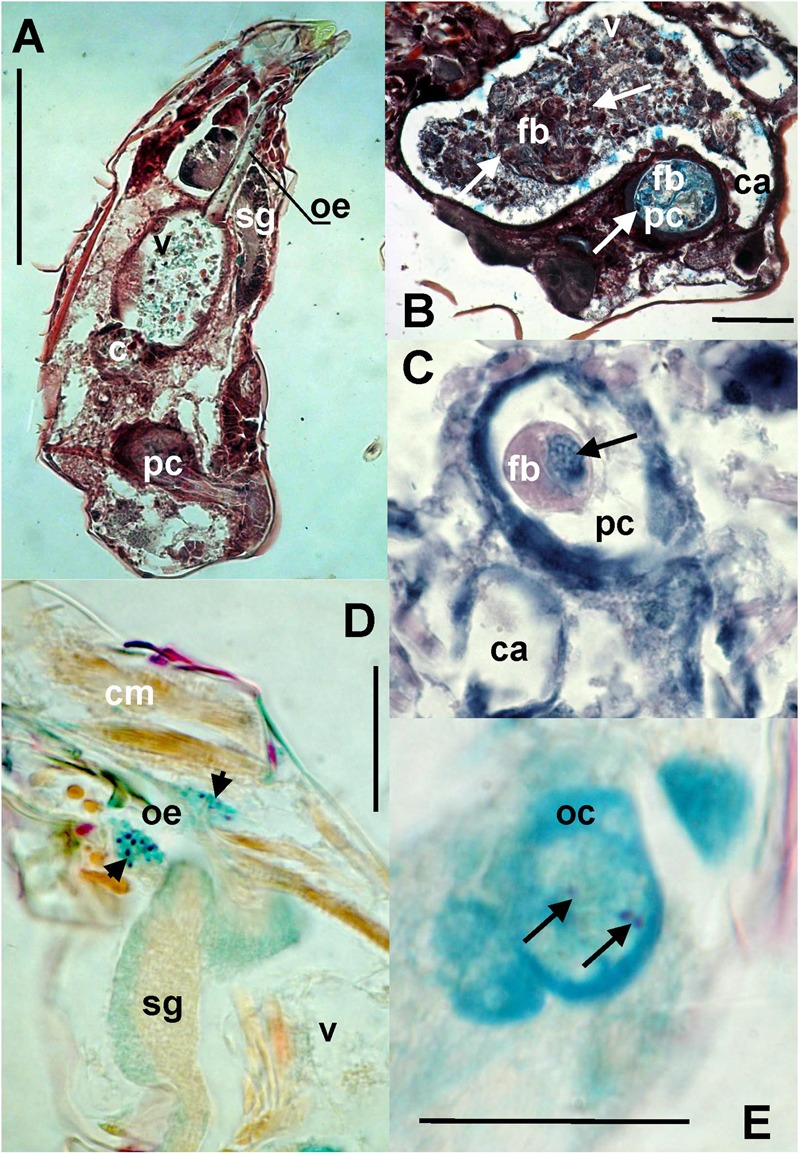
**The images show: **(A)** total view of the digestive tract of *T. putrescentiae*; **(B)** total view of the ventriculus and post-colon with ingested food; arrows point to food boli; **(C)** detail view of the post-colon of specimens with food boli formed from mucoid substances and fragments of diet (arrow); **(D)** detail view of the salivary glands with stained bacterial cells (arrows); **(E)** detail view of oocyte with stained bacterial cells (arrows).** Staining: **(A,B)** Masson’s triple stain, **(C)** Ziehl–Neelsen, **(D,E)** Mann Dominici; Scales: **(A,B)** 100 μm, **(D,E)** 25 μm. c, colon; ca, caecum; cm, cheliceral muscles; fb, food bolus; oc, oocyte; oe, esophagus; pc, post-colon; sg, synganglion; v, ventriculus.

Bacteria were present on the mite surfaces, in bacteryocites, reproductive tracts and salivary glands (**Figure 7D**). The bacteria were not identified in food boli. The observed food boli (**Figure 7A**) contained concentrated mucoid substances or unidentified food fragments (**Figures 7B,C**). However, the mites ingested bacteria randomly, as indicated by the presence of bacteria in the foregut. In the Phillips and Dog populations we found bacteria inside ovaria (**Figure 7E**).

**FIGURE 8:**
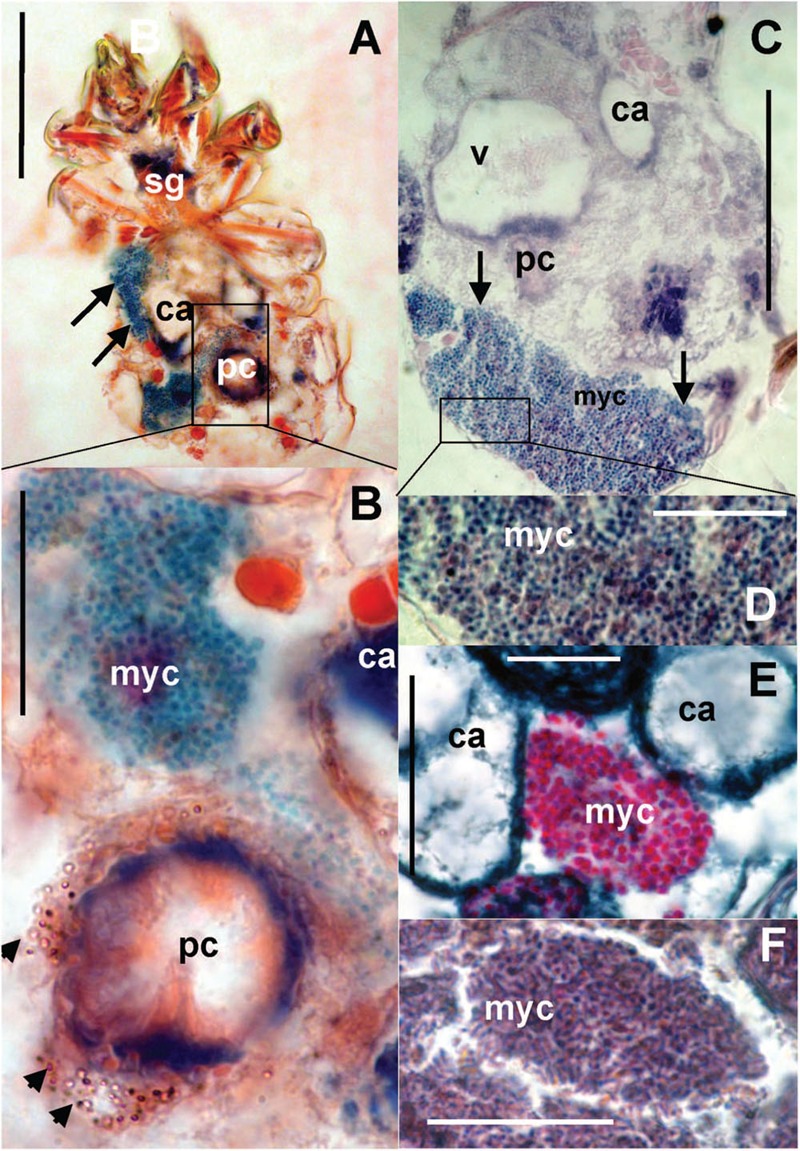
**Histological sections of *T. putrescentiae*: **(A)** Total view of *T. putrescentiae* with bacteriocyte (arrows); **(B)** details of the previous image; the arrows point to guanine crystals near the post-colon; **(C)** sagittal section of the mite body with bacteriocyte (arrows); **(E)** details of the previous image with spherical bacteria; **(F)** localization of bacteriocytes with spherical bacteria, **(F)** details of the bacteriocyte with rod-shaped bacteria.** Staining: **(A,B)** Mann Dominici, **(C,D)** Ziehl–Neelsen, **(E,F)** Masson’s triple stain; Scales. **(A,C)** 100 μm, **(D–F)** 25 μm. ca, caecum; fb, food bolus; myc, bacteriocyte; pc, post-colon; sg, synganglion.

The bacteriocytes (**Figure 8A**) were found in some adults in all observed populations. The bacteria were localized in fat tissues and were of various sizes covering up to one third of the histological sections (**Figure 8C**). The bacteria in the bacteriocytes were formed by spherical (**Figures 8B,D,E**) particles with different staining compared to the rest of tissues; we identified these particles as bacteria. In addition, rod-shaped particles were found in the bacteriocytes in the laboratory population of *T. putrescentiae* (**Figure 8F**).

**FIGURE 9:**
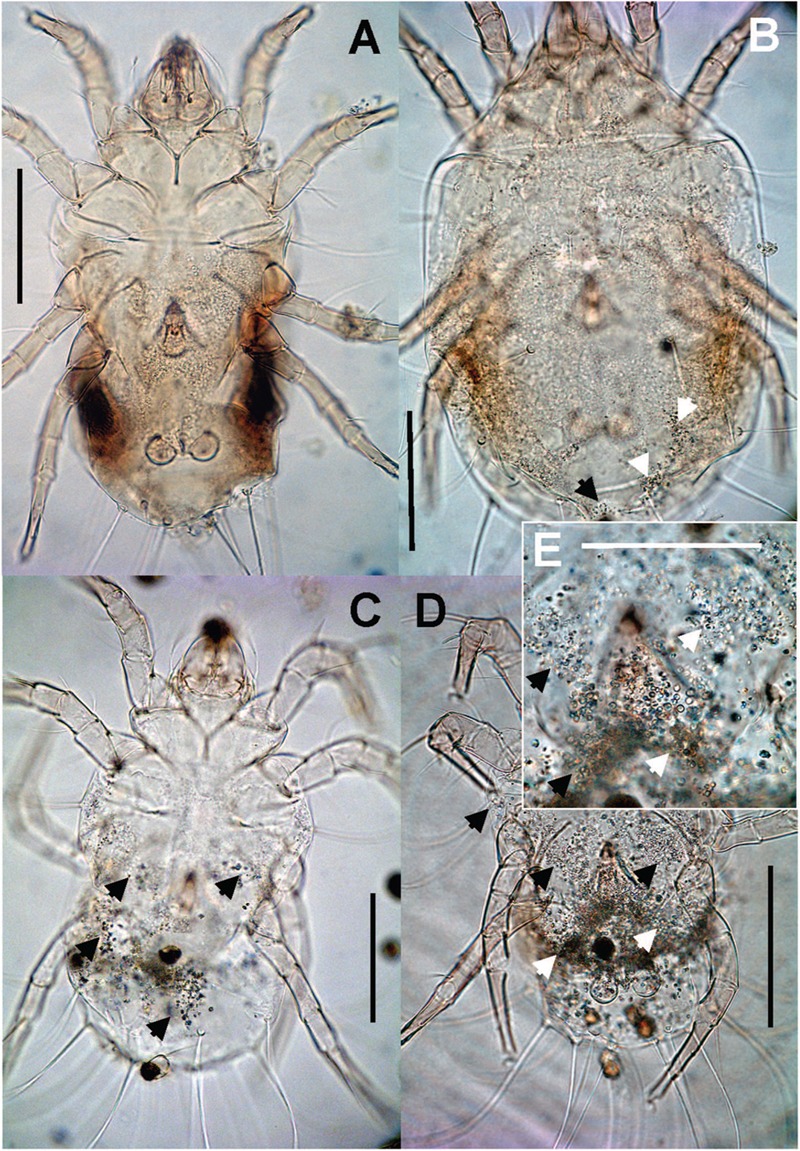
**Guanine deposits in *T. putrescentiae* (mite are mounted on permanent microscopic slides): **(A)** no guanine granules; **(B)** a low number, the granules fill less than 25% of the hysterosoma; **(C)** intermediate, the granules fill 25–50% of the hysterosoma; **(D)** massive, the granules fill more than 50% of the hysterosoma, **(E)** detail of the previous image.** Scales: **(A–D**) 100 μm, **(E)** 25 μm.

**FIGURE 10:**
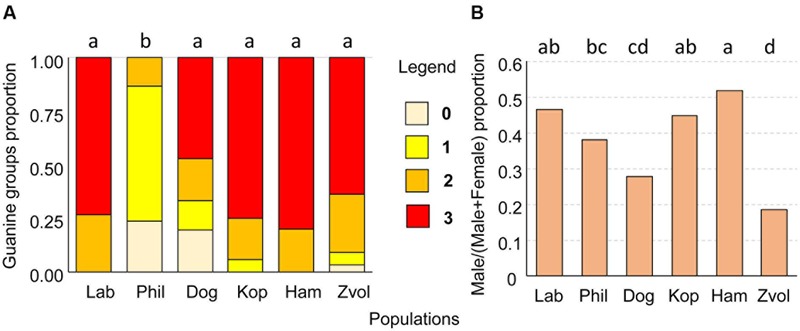
**(A)** The proportion of guanine waste product granules in *T. putrescentiae*; **(B)** comparison of sex ratios, male/(male+female) in examined populations of *T. putrescentiae*. Statistically significant differences (α = 0.05) in multiple comparisons are indicated by different letters. Abbreviations for the *T. putrescentiae* populations are listed in **Table 1**. (0) no guanine granules, (1) low number, granules fill less than 25% of the hysterosoma, (2) intermediate, granules fill between 25 and 50% of the hysterosoma, and (3) massive, granules fill more than 50% of the hysterosoma.

The guanin granulae filled hysterosoma of mites with different intensity (**Figure 9**). The populations significantly differed (Chi-square = 119.37, *P* < 0.001) in the guanine contents in their hysterosoma (**Figure 10A**). The differences were between Phillips population with prevailing low guanine contents and the rest of populations with high guanine contents (**Figure 9**).

### Estimation of Sex Ratio

Males were present in all studied populations. As estimated under a compound microscope using slide-mounted mites, proportions male/(male+female) differed among the *T. putrescentiae* populations (Chi-square = 72.63, *P* < 0.001) formed following groups: (i) proportion about 0.5: Laboratory, Koppert, and Ham; (ii) female prevailing populations: Phillips, Dog, and Zvoleneves populations (**Figure 10B**).

## Discussion

We found significant differences in bacterial communities among various *T. putrescentiae* populations using Sanger sequencing of cloned bacterial 16S rRNA amplicons. Because of the complex taxonomy of *T. putrescentiae* and a possibility of the presence of cryptic species, we inferred single-gene phylogenetic trees aimed at species identification and analysis of sequences available in GenBank. We sequenced two genes most commonly used for *Tyrophagus*, CO1 and ITS-2. Both generated phylogenetic trees showed the presence of two distinct species, *T. putrescentiae* and *T. fanetzhangorum. T. putrescentiae* was present in five populations except. Zvoleneves population was a mixure of *T. fanetzhangorum* and *T. putrescentiae*. These analyses also allowed classification of previously unidentified GenBank sequences (**Figures 1** and **2**). We did not conduct a combined analysis of the two genes because many sequences from GenBank are available for one but not for both of these genes. We suggest that in the case of Zvoleneves population might be the results of bacterial community influenced by an additional factor, that is, the presence of *T. fanetzhangorum* having its own unique bacterial community.

The differences in bacterial communities confirm the previous results: there is clear variation in bacterial communities among different populations of mites, and this variation may be due to environmental factors such as differences in the diet, micro-habitat or simply the geographic sources, as shown previously for *Rhizoglyphus robini* (Zindel et al., 2013) and *Carpoglpyhus lactis* (Hubert et al., 2015). The bacterial community of *T. putrescentiae* was formed mainly by symbiotic or parasitic bacteria, and contained previously identified bacteria including *Bartonella*-like, *Cardinium* (Kopecky et al., 2013, 2014b), and *Wolbachia* (Brown and Lloyd, 2015). A *Blattabacterium*-like symbiont and *Solitalea*-like bacteria were newly identified for *T. putrescentiae*. The presence of *Cardinium, Wolbachia, Solitalea*-like bacteria and *Blattabacterium*-like bacteria in the eggs of some of the compared *T. putrescentiae* populations indicate symbiotic or parasitic association between these bacteria and *T. putrescentiae*. It also indicates mother to offspring (vertical) transmission via eggs of these bacteria. In one case (Phillips population), we found *Bartonella* in the eggs; however, in four *T. putrescentiae* populations, *Bartonella* was not observed in the eggs, suggesting that *Bartonella* is not vertically transmitted.

The next group of identified bacteria, i.e., *Bacillus, Moraxella, Staphylococcus, Kocuria*, and *Microbacterium* could have originated from ingested diet (Hubert et al., 2012a,b; Kopecky et al., 2014a,b). We suggest that ingested bacteria serve as source of nutrients for the mites whether by direct food source (Erban and Hubert, 2008) and/or that that they predigest the food with exo-enzymes (Erban et al., 2016).

The interesting finding is the presence of *Solitalea*-like bacteria, which we identified previously in cloned 16S rRNA sequences from a laboratory strain of the grain mite *A. siro* (Hubert et al., 2012a,b). However, one sequence of *Pedobacter* (*Solitalea*-like) was found in a Chinese *T. putrescentiae* population from edible fungi by Qu et al. (2015). *Solitalea*-like bacteria were detected in this study in all the *T. putrescentiae* populations. Recently, we detected *Solitalea*-like bacteria in the reproductive tract and parenchymal tissues of *A. siro* (Hubert et al., under review). The bacteria were also present in the eggs (Hubert et al., under review). Given these findings, we suggest that, similarly to *Cardinium*, this *Solitalea*-like bacteria might be either symbiotic or parasitic.

*Wolbachia* are well-known bacteria in insects and mites (Glowska et al., 2015); however, only a few such studies are available for Astigmata. A recent study demonstrated that *T. putrescentiae* mites feeding on *Drosophila* corpses, including *Wolbachia*-infected corpses, are possible vectors of *Wolbachia* (Brown and Lloyd, 2015). In this study, we found that the *Wolbachia* sequences from *T. putrescentiae* are more similar to *Wolbachia* in quill mites *Torotrogla cardueli* (Acari: Syringophilidae; Glowska et al., 2015), aphids (Augustinos et al., 2011), and nematodes (Haegeman et al., 2009). We also found that the *Wolbachia* sequences from Dog and Phillips *T. putrescentiae* populations clustered altogether suggesting their high similarity. *Wolbachia* manipulates sexual reproductions in mites, causing cytoplasmic incompatibility and feminization of males (Breeuwer and Jacobs, 1996). The results for sex ratio observed in both populations of the *T. putrescentiae* where we identified *Wolbachia* indicated that males were present. At the population level, *T. putrescentiae* is infested by at least two bacteria with the known ability to manipulate sexual reproduction in their hosts, i.e., *Cardinium* and *Wolbachia*. However, our sampling method did not provide information about the distribution of bacteria among individual mites. We localized the bacteria in oocytes and the bacteria were not observed in all individuals. This means that the infection spreads in a mite population with some dynamics. The described horizontal transfer of *Wolbachia* from *Drosophila* to *T. putrescentiae* (Brown and Lloyd, 2015) might suggest a possibility for mite-to-mite transfer also in our system. However, infection in other species of astigmatid mites in laboratory populations was not observed (Kopecky et al., 2014a), and the results obtained here using *Wolbachia-*specific primers were similar (see **Table 2**). In addition, in the populations infected with *Wolbachia*, a lower proportion of mite males was observed.

*Blattabacterium* is a member of Flavobacteriales (Bacteroidetes), which are obligate mutualistic endosymbiotic bacteria living in the fat bodies of cockroaches (Sabree et al., 2009). The association between cockroaches and this bacterium allows cockroaches to successfully subsist on nitrogen-poor diets and exploit nitrogenous waste (Sabree et al., 2009). Other related taxa are symbionts of sap-feeding insects, i.e., Hemiptera: Diaspididae, Cicadellidae, Coccoidea (Moran et al., 2005; Gruwell et al., 2007; Rosenblueth et al., 2012). Here, we found a related bacterium in the population found on dog food. Dog food is designed for carnivorous mammals and contains high fat and nitrogen, which makes it very suitable for the rapid growth and development of *T. putrescentiae* (Rybanska et al., 2015). Acquiring such a bacterium for a *T. putrescentiae* population that infests dry dog food seems to be beneficial to *T. putrescentiae*, but the exact nature of mite and bacterium interactions is unknown.

The *Blattabacterium* and *Bartonella-*relative taxa are known to influence nitrogen metabolisms of their hosts (van Borm et al., 2002; Sabree et al., 2009). Because we found such bacteria in *T. putrescentiae*, we focused on the comparison of guanine contents in specimens in the analyzed populations. We hypothesized that *Blattabacterium*-like bacteria reduce nitrogen waste in the parenchymal tissue of *T. putrescentiae* in the same way as suggested for cockroaches (Sabree et al., 2009). Guanine is the waste product of nitrogenous metabolisms (Levinson et al., 1991a). It is present in feces and has a kairomone function as was observed in the model species *A. siro* (Levinson et al., 1991b). *T. putrescentiae* has the ability to accumulate guanine in the parenchymal tissues (Smrz and Catska, 1989). It was documented that *T. putrescentiae* on nitrogenous-rich diets, such as some fungi, accumulate guanine in the fat tissues (Smrz and Catska, 1989; Smrz, 2003). Massive feeding on a fungal-type diet leads to an irreversible accumulation of guanine called “white body syndrome,” in which guanine crystals form in large numbers in fat tissues and suppress the internal organ functions of mites, leading to a damage (Smrz and Catska, 1989). But the categorization of guanine granules in mite bodies did not confirm this suggestion, because low guanine contents were observed in Phillips not in Dog populations. The high guanine content in our populations of *T. putrescentiae* is a possible result of nitrogen un-balanced diets when the mites are not able to eliminate nitrogenous waste (Smrz, 2003).

Bacteriocytes were formed by bacteria in mite specimens that consume a fungal diet (Smrz and Catska, 1989). It was suggested that bacteria can enter from the gut to the bacteriocytes in the fat tissues and participate in chitin digestion (Smrz and Trelova, 1995; Smrz, 2003). In this study, we found bacteriocytes in specimens from all populations, but bacteriocytes were not present in all specimens. We did not identify bacteria forming bacteriocytes, although only *Solitalea* was identified in all populations. However, previously we observed *Solitalea-*like bacteria in *A. siro*, and bacteriocytes were not present (Hubert et al., 2012a).

We hypothesize that the habitat and food plasticity exhibited by *T. putrescentiae* is mediated by different bacterial communities associated with mite populations. Of the 35 identified OTUs, only *Solitalea* was shared by all populations, indicating that different populations of *T. putrescentiae* differ in their bacterial communities. Previously, large changes in bacterial communities were observed after a diet switch in *T. putrescentiae*. However, those differences were due to bacterial taxa ingested with the diet, i.e., *Bacillaceae* (*Bacillus, Lysinibacillus, Oceanobacillus*, and *Virgibacillus*) and *Micrococcales* (*Kocuria* and *Brevibacterium*; Hubert et al., 2012b; Kopecky et al., 2014b). This was also supported by observations of a field strain of *T. putrescentiae* in laboratory experiments on fungal diets, when *Alcaligenes faecalis, Agrobacterium* sp., *Serratia marcescens*, and *Achromobacter* sp. were identified by plating and cultivation of bacteria obtained from the homogenates of *T. putrescentiae* specimens that had consumed the fungi (Smrz et al., 1991; Smrz and Soukalova, 2008; Smrz and Catska, 2010). Here, we observed changes in *T. putrescentiae* populations in the presence and infestation rates of *Bacillus, Staphylococcus, Kocuria*, and *Moraxella* as indicated by sequencing of cloned 16S rRNA sequences. Among these, *Moraxella* was associated with a mite population feeding on ham. Bacteria forming food boli were not observed in the studied populations of *T. putrescentiae*. One explanation is that these bacteria are ingested in low numbers along with fragments of fungal mycelium or plant debris.

From a practical point of view, mites are important pests, and mite-caused damage increases as the population density increases. It is well-known that different food sources influence enzyme physiology of mites resulting in differences in population growths (Smrz and Catska, 1987; Erban and Hubert, 2008, 2010; Erban et al., 2009, 2016; Nesvorna et al., 2012; Rybanska et al., 2015). The results of this study indicate that diet and habitats influence not only the ingested spectrum of bacteria but also the symbiotic and parasitic taxa. These two components of the bacterial community can affect both mite fitness and population growth, which result in variability in the growth of various populations, causing higher interspecies variability than variability among species (Bowman, 1984). Therefore, the results of this study are also important for understanding nutritional biology of mites.

## Author Contributions

JH, TE, PK, JS, and TP: Scientific writing; JK: bioinformatics; MN: experiments, molecular biology, JH, JS and TE: experimental design, PK: taxonomy and interpretation, JH, and JS: microanatomy.

## Conflict of Interest Statement

The authors declare that the research was conducted in the absence of any commercial or financial relationships that could be construed as a potential conflict of interest.

## References

[B1] Altschul S. F. Madden T. L. Schaffer A. A. Zhang J. Zhang Z. Miller W. (1997). Gapped BLAST and PSI-BLAST: a new generation of protein database search programs. *Nucleic Acids Res.* 25 3389–3402. 10.1093/nar/25.17.3389 9254694 PMC146917

[B2] Ashelford K. E. Chuzhanova N. A. Fry J. C. Jones A. J. Weightman A. J. (2005). At least 1 in 20 16S rRNA sequence records currently held in public repositories is estimated to contain substantial anomalies. *Appl. Environ. Microbiol.* 71 7724–7736. 10.1128/AEM.71.12.7724-7736.2005 16332745 PMC1317345

[B3] Ashelford K. E. Chuzhanova N. A. Fry J. C. Jones A. J. Weightman A. J. (2006). New screening software shows that most recent large 16S rRNA gene clone libraries contain chimeras. *Appl. Environ. Microbiol.* 72 5734–5741. 10.1128/AEM.00556-06 16957188 PMC1563593

[B4] Augustinos A. A. Santos-Garcia D. Dionyssopoulou E. Moreira M. Papapanagiotou A. Scarvelakis M. (2011). Detection and characterization of *Wolbachia* infections in natural populations of aphids: is the hidden diversity fully unraveled? *PLoS ONE* 6:e28695 10.1371/journal.pone.0028695 PMC3236762 22174869

[B5] Barbieri E. Paster B. J. Hughes D. Zurek L. Moser D. P. Teske A. (2001). Phylogenetic characterization of epibiotic bacteria in the accessory nidamental gland and egg capsules of the squid *Loligo pealei* (Cephalopoda: Loliginidae). *Environ. Microbiol.* 3 151–167. 10.1046/j.1462-2920.2001.00172.x 11321532

[B6] Beroiz B. Couso-Ferrer F. Ortego F. Chamorro M. J. Arteaga C. Lombardero M. (2014). Mite species identification in the production of allergenic extracts for clinical use and in environmental samples by ribosomal DNA amplification. *Med. Vet. Entomol.* 28 287–296. 10.1111/mve.12052 24617319

[B7] Bochkov A. V. Klimov P. B. Hestvik G. Saveljev A. P. (2014). Integrated Bayesian species delimitation and morphological diagnostics of chorioptic mange mites (Acariformes: Psoroptidae: Chorioptes). *Parasitol. Res.* 113 2603–2627. 10.1007/s00436-014-3914-9 24820039

[B8] Bowman C. E. (1984). “Comparative enzymology of economically important astigmatid mites,” in *Acarology VI* Vol. 2 eds Griffiths D. A. Bowman C. E. (Chichester: Ellis Horwood) 993–1001.

[B9] Breeuwer J. A. J. Jacobs G. (1996). *Wolbachia*: intracellular manipulators of mite reproduction. *Exp. Appl. Acarol.* 20 421–434. 10.1007/BF00053306 8856963

[B10] Brown A. N. Lloyd V. K. (2015). Evidence for horizontal transfer of *Wolbachia* by a *Drosophila* mite. *Exp. Appl. Acarol.* 66 301–311. 10.1007/s10493-015-9918-z 25921489

[B11] Clark J. W. Kambhampati S. (2003). Phylogenetic analysis of *Blattabacterium*, endosymbiotic bacteria from the wood roach, *Cryptocercus* (Blattodea: Cryptocercidae), including a description of three new species. *Mol. Phylogenet. Evol.* 26 82–88. 10.1016/S1055-7903(02)00330-5 12470940

[B12] Colloff M. J. (2009). *Dust Mites.* Dordrecht: Springer 10.1007/978-90-481-2224-0

[B13] Darriba D. Taboada G. L. Doallo R. Posada D. (2012). jModelTest 2: more models, new heuristics and parallel computing. *Nat. Methods* 9 772–772. 10.1038/nmeth.2109 22847109 PMC4594756

[B14] Dermauw W. Van Leeuwen T. Vanholme B. Tirry L. (2009). The complete mitochondrial genome of the house dust mite *Dermatophagoides pteronyssinus* (Trouessart): a novel gene arrangement among arthropods. *BMC Genomics* 10:107 10.1186/1471-2164-10-107 PMC2680895 19284646

[B15] Dillon R. J. Dillon V. M. (2004). The gut bacteria of insects: nonpathogenic interactions. *Annu. Rev. Entomol.* 49 71–92. 10.1146/annurev.ento.49.061802.123416 14651457

[B16] Douglas A. E. (2009). The microbial dimension in insect nutritional ecology. *Funct. Ecol.* 23 38–47. 10.1111/j.1365-2435.2008.01442.x

[B17] Douglas A. E. (2015). Multiorganismal insects: diversity and function of resident microorganisms. *Annu. Rev. Entomol.* 60 17–34. 10.1146/annurev-ento-010814-020822 25341109 PMC4465791

[B18] Duek L. Kaufman G. Palevsky E. Berdicevsky I. (2001). Mites in fungal cultures. *Mycoses* 44 390–394. 10.1046/j.1439-0507.2001.00684.x 11766104

[B19] Erban T. Erbanova M. Nesvorna M. Hubert J. (2009). The importance of starch and sucrose digestion in nutritive biology of synanthropic acaridid mites: alpha-amylases and alpha-glucosidases are suitable targets for inhibitor-based strategies of mite control. *Arch. Insect Biochem. Physiol.* 71 139–158. 10.1002/arch.20312 19480003

[B20] Erban T. Hubert J. (2008). Digestive function of lysozyme in synanthropic acaridid mites enables utilization of bacteria as a food source. *Exp. Appl. Acarol.* 44 199–212. 10.1007/s10493-008-9138-x 18357505

[B21] Erban T. Hubert J. (2010). Comparative analyses of proteolytic activities in seven species of synanthropic acaridid mites. *Arch. Insect Biochem. Physiol.* 75 187–206. 10.1002/arch.20388 20936642

[B22] Erban T. Rybanska D. Harant K. Hortova B. Hubert J. (2016). Feces derived allergens of *Tyrophagus putrescentiae* reared on dried dog food and evidence of the strong nutritional interaction between the mite and *Bacillus cereus* producing protease bacillolysins and exo-chitinases. *Front. Physiol.* 7:53 10.3389/fphys.2016.00053 PMC4764834 26941650

[B23] Erban T. Rybanska D. Hubert J. (2015). Population growth of the generalist mite *Tyrophagus putrescentiae* (Acari: Acaridida) following adaptation to high- or low-fat and high- or low-protein diets and the effect of dietary switch. *Environ. Entomol.* 44 1599–1604. 10.1093/ee/nvv129 26314031

[B24] Franz J.-T. Masuch G. Musken H. Bergmann K.-C. (1997). Mite fauna of German farms. *Allergy* 52 1233–1237. 10.1111/j.1398-9995.1997.tb02529.x 9450144

[B25] Garcia N. (2004). Efforts to control mites on Iberian ham by physical methods. *Exp. Appl. Acarol.* 32 41–50. 10.1023/B:APPA.0000018165.80420.c9 15139271

[B26] Ge M.-K. Sun E.-T. Jia C.-N. Kong D.-D. Jiang Y.-X. (2014). Genetic diversity and differentiation of *Lepidoglyphus destructor* (Acari: Glycyphagidae) inferred from inter-simple sequence repeat (ISSR) fingerprinting. *Syst. Appl. Acarol.* 19 491–498. 10.11158/saa.19.4.12

[B27] Glowska E. Dragun-Damian A. Dabert M. Gerth M. (2015). New *Wolbachia* supergroups detected in quill mites (Acari: Syringophilidae). *Infect. Genet. Evol.* 30 140–146. 10.1016/j.meegid.2014.12.019 25541519

[B28] Gruwell M. E. Hardy N. B. Gullan P. J. Dittmar K. (2010). Evolutionary relationships among primary endosymbionts of the mealybug subfamily Phenacoccinae (Hemiptera: Coccoidea: Pseudococcidae). *Appl. Environ. Microbiol.* 76 7521–7525. 10.1128/AEM.01354-10 20851962 PMC2976180

[B29] Gruwell M. E. Morse G. E. Normark B. B. (2007). Phylogenetic congruence of armored scale insects (Hemiptera: Diaspididae) and their primary endosymbionts from the phylum Bacteroidetes. *Mol. Phylogenet. Evol.* 44 267–280. 10.1016/j.ympev.2007.01.014 17400002

[B30] Guindon S. Dufayard J.-F. Lefort V. Anisimova M. Hordijk W. Gascuel O. (2010). New algorithms and methods to estimate maximum-likelihood phylogenies: assessing the performance of PhyML 3.0. *Syst. Biol.* 59 307–321. 10.1093/sysbio/syq010 20525638

[B31] Guindon S. Gascuel O. (2003). A simple, fast, and accurate algorithm to estimate large phylogenies by maximum likelihood. *Syst. Biol.* 52 696–704. 10.1080/10635150390235520 14530136

[B32] Haegeman A. Vanholme B. Jacob J. Vandekerckhove T. T. Claeys M. Borgonie G. (2009). An endosymbiotic bacterium in a plant-parasitic nematode: member of a new *Wolbachia* supergroup. *Int. J. Parasitol.* 39 1045–1054. 10.1016/j.ijpara.2009.01.006 19504759

[B33] Hammer O. Harper D. A. T. Ryan P. D. (2001). *PAST: Paleontological Statistics Software Package for Education and Data Analysis. Palaeontol. Electron. 4:4*. Available at: http://palaeo-electronica.org/2001_1/past/issue1_01.htm [accessed June 10, 2016].

[B34] Hoy M. A. Jeyaprakash A. (2005). Microbial diversity in the predatory mite *Metaseiulus* occidentalis (Acari: Phytoseiidae) and its prey, *Tetranychus urticae* (Acari: Tetranychidae). *Biol. Control* 32 427–441. 10.1016/j.biocontrol.2004.12.012

[B35] Hubert J. Kopecky J. Perotti M. A. Nesvorna M. Braig H. R. Sagova-Mareckova M. (2012a). Detection and identification of species-specific bacteria associated with synanthropic mites. *Microb. Ecol.* 63 919–928. 10.1007/s00248-011-9969-6 22057398

[B36] Hubert J. Nesvorna M. Kopecky J. Sagova-Mareckova M. Poltronieri P. (2015). *Carpoglyphus lactis* (Acari: Astigmata) from various dried fruits differed in associated micro-organisms. *J. Appl. Microbiol.* 118 470–484. 10.1111/jam.12714 25469657

[B37] Hubert J. Nesvorna M. Sagova-Mareckova M. Kopecky J. (2012b). Shift of bacterial community in synanthropic mite *Tyrophagus putrescentiae* induced by *Fusarium* fungal diet. *PLoS ONE* 7:e48429 10.1371/journal.pone.0048429 PMC3485207 23119013

[B38] Hubert J. Stejskal V. Munzbergova Z. Kubatova A. Vanova M. Zdarkova E. (2004). Mites and fungi in heavily infested stores in the Czech Republic. *J. Econ. Entomol.* 97 2144–2153. 10.1093/jee/97.6.2144 15666776

[B39] Hubert J. Sustr V. Smrz J. (1999). Feeding of the oribatid mite *Scheloribates laevigatus* (Acari: Oribatida) in laboratory experiments. *Pedobiologia* 43 328–339.

[B40] Hughes A. M. (1976). *The Mites of Stored Food and Houses Volume 9 of Technical bulletin (Great Britain. Ministry of Agriculture, Fisheries and Food)* 2nd Edn London: Her Majesty’s Stationery Office.

[B41] Klimov P. B. OConnor B. (2013). Is permanent parasitism reversible?—critical evidence from early evolution of house dust mites. *Syst. Biol.* 62 411–423. 10.1093/sysbio/syt008 23417682

[B42] Klimov P. B. OConnor B. M. (2008). Origin and higher-level relationships of psoroptidian mites (Acari: Astigmata: Psoroptidia): evidence from three nuclear genes. *Mol. Phylogenet. Evol.* 47 1135–1156. 10.1016/j.ympev.2007.12.025 18289886

[B43] Klimov P. B. OConnor B. M. (2009a). Conservation of the name *Tyrophagus putrescentiae*, a medically and economically important mite species (Acari: Acaridae). *Int. J. Acarol.* 35 95–114. 10.1080/01647950902902587

[B44] Klimov P. B. OConnor B. M. (2009b). Improved tRNA prediction in the American house dust mite reveals widespread occurrence of extremely short minimal tRNAs in acariform mites. *BMC Genomics* 10:598 10.1186/1471-2164-10-598 PMC2797822 20003349

[B45] Kopecky J. Nesvorna M. Hubert J. (2014a). *Bartonella*-like bacteria carried by domestic mite species. *Exp. Appl. Acarol.* 64 21–32. 10.1007/s10493-014-9811-1 24711066

[B46] Kopecky J. Nesvorna M. Mareckova-Sagova M. Hubert J. (2014b). The effect of antibiotics on associated bacterial community of stored product mites. *PLoS ONE* 9:e112919 10.1371/journal.pone.0112919 PMC4227874 25387104

[B47] Kopecky J. Perotti M. A. Nesvorna M. Erban T. Hubert J. (2013). *Cardinium* endosymbionts are widespread in synanthropic mite species (Acari: Astigmata). *J. Invertebr. Pathol.* 112 20–23. 10.1016/j.jip.2012.11.001 23147105

[B48] Kramar J. (1953). The contribution to microscopic preparation of Arthropods. [Prispevek k mikroskopicke preparaci clenovcu.]. *Ceskoslov. Biol.* 2 57–58.

[B49] Lane D. J. (1991). “16S/23S rRNA sequencing,” in *Nucleic Acid Techniques in Bacterial Systematics* eds Stackebrandt E. Goodfellow M. (New York, NY, USA: John Wiley and Sons) 115–175.

[B50] Lartillot N. Lepage T. Blanquart S. (2009). PhyloBayes 3: a Bayesian software package for phylogenetic reconstruction and molecular dating. *Bioinformatics* 25 2286–2288. 10.1093/bioinformatics/btp368 19535536

[B51] Levinson H. Z. Levinson A. R. Muller K. (1991a). Functional adaption of two nitrogenous waste products in evoking attraction and aggregation of flour mites (*Acarus siro* L.). *Anz. Schadlingskd. Pfl. Umwelt.* 64 55–60. 10.1007/bf01909743

[B52] Levinson H. Z. Levinson A. R. Muller K. (1991b). The adaptive function of ammonia and guanine in the biocoenotic association between Ascomycetes and flour mites (*Acarus siro* L.). *Naturwissenschaften* 78 174–176. 10.1007/bf01136207

[B53] Liu Y. C. Chang S. C. Chen W. H. Shu W. B. (2006). The application of single-step nested multiplex polymerase chain reaction for the identification of *Rhizoglyphus echinopus, R. robini and R. setosus* simultaneously. *Plant Prot. Bull.* 48 101–116.

[B54] Meeus I. Vercruysse V. Smagghe G. (2012). Molecular detection of *Spiroplasma apis* and *Spiroplasma melliferum* in bees. *J. Invertebr. Pathol.* 109 172–174. 10.1016/j.jip.2011.11.006 22138255

[B55] Moran N. A. Tran P. Gerardo N. M. (2005). Symbiosis and insect diversification: an ancient symbiont of sap-feeding insects from the bacterial phylum Bacteroidetes. *Appl. Environ. Microbiol.* 71 8802–8810. 10.1128/AEM.71.12.8802-8810.2005 16332876 PMC1317441

[B56] Nesvorna M. Gabrielova L. Hubert J. (2012). Suitability of a range of *Fusarium* species to sustain populations of three stored product mite species (Acari: Astigmata). *J. Stored Prod. Res.* 48 37–45. 10.1016/j.jspr.2011.08.006

[B57] Noge K. Mori N. Tanaka C. Nishida R. Tsuda M. Kuwahara Y. (2005). Identification of astigmatid mites using the second internal transcribed spacer (ITS2) region and its application for phylogenetic study. *Exp. Appl. Acarol.* 35 29–46. 10.1007/s10493-004-1953-0 15776999

[B58] OConnor B. M. (1979). “Evolutionary origins of astigmatid mites inhabiting stored products,” in *Proceedings of the V International Congress of Acarology: Recent Advances in Acarology, Volume 1, August 6–12, 1978 Michigan State University, East Lansing* ed. Rodriguez G. J. (New York, NY: Academic Press) 273–278. 10.1016/b978-0-12-592201-2.50038-5

[B59] OConnor B. M. (1982). Evolutionary ecology of astigmatid mites. *Annu. Rev. Entomol.* 27 385–409. 10.1146/annurev.en.27.010182.002125

[B60] O’Neill S. L. Giordano R. Colbert A. M. Karr T. L. Robertson H. M. (1992). 16S rRNA phylogenetic analysis of the bacterial endosymbionts associated with cytoplasmic incompatibility in insects. *Proc. Natl. Acad. Sci. U.S.A.* 89 2699–2702. 10.1073/pnas.89.7.2699 1557375 PMC48729

[B61] Palyvos N. E. Emmanouel N. G. Saitanis C. J. (2008). Mites associated with stored products in Greece. *Exp. Appl. Acarol.* 44 213–226. 10.1007/s10493-008-9145-y 18379887

[B62] Pruesse E. Peplies J. Glockner F. O. (2012). SINA: accurate high-throughput multiple sequence alignment of ribosomal RNA genes. *Bioinformatics* 28 1823–1829. 10.1093/bioinformatics/bts252 22556368 PMC3389763

[B63] Qu S.-X. Li H.-P. Ma L. Hou L.-J. Lin J.-S. Song J.-D. (2015). Effects of different edible mushroom hosts on the development, reproduction and bacterial community of *Tyrophagus putrescentiae* (Schrank). *J. Stored Prod. Res.* 61 70–75. 10.1016/j.jspr.2014.12.003

[B64] Quast C. Pruesse E. Yilmaz P. Gerken J. Schweer T. Yarza P. (2013). The SILVA ribosomal RNA gene database project: improved data processing and web-based tools. *Nucleic Acids Res.* 41 D590–D596. 10.1093/nar/gks1219 23193283 PMC3531112

[B65] Robertson P. L. (1961). A morphological study of variation in *Tyrophagus* (Acarina), with particular reference to populations infesting cheese. *Bull. Entomol. Res.* 52 501–529. 10.1017/s0007485300055565 14492832

[B66] Rosenblueth M. Sayavedra L. Samano-Sanchez H. Roth A. Martinez-Romero E. (2012). Evolutionary relationships of flavobacterial and enterobacterial endosymbionts with their scale insect hosts (Hemiptera: Coccoidea). *J. Evol. Biol.* 25 2357–2368. 10.1111/j.1420-9101.2012.02611.x 22994649

[B67] Rozej E. Witalinski W. Szentgyorgyi H. Wantuch M. Moron D. Woyciechowski M. (2012). Mite species inhabiting commercial bumblebee (*Bombus terrestris*) nests in Polish greenhouses. *Exp. Appl. Acarol.* 56 271–282. 10.1007/s10493-012-9510-8 22270110 PMC3273685

[B68] Russell J. A. Moreau C. S. Goldman-Huertas B. Fujiwara M. Lohman D. J. Pierce N. E. (2009). Bacterial gut symbionts are tightly linked with the evolution of herbivory in ants. *Proc. Natl. Acad. Sci. U.S.A.* 106 21236–21241. 10.1073/pnas.0907926106 19948964 PMC2785723

[B69] Rybanska D. Hubert J. Markovic M. Erban T. (2015). Dry dog food integrity and mite strain influence the density-dependent growth of the stored-product mite *Tyrophagus putrescentiae* (Acari: Acaridida). *J. Econ. Entomol.* 109 454–460. 10.1093/jee/tov298 26476559

[B70] Sabree Z. L. Kambhampati S. Moran N. A. (2009). Nitrogen recycling and nutritional provisioning by *Blattabacterium*, the cockroach endosymbiont. *Proc. Natl. Acad. Sci. U.S.A.* 106 19521–19526. 10.1073/pnas.0907504106 19880743 PMC2780778

[B71] Schloss P. D. Westcott S. L. Ryabin T. Hall J. R. Hartmann M. Hollister E. B. (2009). Introducing mothur: open-source, platform-independent, community-supported software for describing and comparing microbial communities. *Appl. Environ. Microbiol.* 75 7537–7541. 10.1128/AEM.01541-09 19801464 PMC2786419

[B72] Smrz J. (1989). Internal anatomy of *Hypochthonius rufulus* (Acari: Oribatida). *J. Morphol.* 200 215–230. 10.1002/jmor.1052000210 29865659

[B73] Smrz J. (2003). Microanatomical and biological aspects of bacterial associations in *Tyrophagus putrescentiae* (Acari: Acaridida). *Exp. Appl. Acarol.* 31 105–113. 10.1023/b:appa.0000005111.05959.d6 14756405

[B74] Smrz J. Catska V. (1987). Food selection of the field population of *Tyrophagus putrescentiae* (Schrank) (Acari. Acarida). *J. Appl. Entomol.* 104 329–335. 10.1111/j.1439-0418.1987.tb00533.x

[B75] Smrz J. Catska V. (1989). The effect of the consumption of some soil fungi on the internal microanatomy of the mite *Tyrophagus putrescentiae* (Schrank) (Acari. Acaridida). *Acta Univ. Carol. Biol.* 33 81–93.

[B76] Smrz J. Catska V. (2010). Mycophagous mites and their internal associated bacteria cooperate to digest chitin in soil. *Symbiosis* 52 33–40. 10.1007/s13199-010-0099-6

[B77] Smrz J. Jungova E. (1989). The ecology of a field population of *Tyrophagus putrescentiae* (Acari. Acaridida). *Pedobiologia* 33 183–192.

[B78] Smrz J. Soukalova H. (2008). “Mycophagous mites (Acari: Oribatida and Acaridida) and their cooperation with chitinolytic bacteria,” in *Proceedings of the Sixth European Congress: Integrative Acarology, 21-25 July 2008, Montpellier* eds Bertrand M. Kreiter S. McCoy K. D. Migeon A. Navajas M. Tixier M.-S. (Montpellier: European Association of Acarologists (EURAAC)) 374–377.

[B79] Smrz J. Svobodova J. Catska V. (1991). Synergetic participation of *Tyrophagus putrescentiae* (Schrank) (Acari; Acaridida) and its associated bacteria on the destruction of some soil micromycetes. *J. Appl. Entomol.* 111 206–210. 10.1111/j.1439-0418.1991.tb00312.x

[B80] Smrz J. Trelova M. (1995). The association of bacteria and some soil mites (Acari: Oribatida and Acaridida). *Acta Zool. Fenn.* 196 120–123.

[B81] Solarz K. Senczuk L. Maniurka H. Cichecka E. Peszke M. (2007). Comparisons of the allergenic mite prevalence in dwellings and certain outdoor environments of the Upper Silesia (southwest Poland). *Int. J. Hyg. Environ. Health* 210 715–724. 10.1016/j.ijheh.2006.11.007 17222584

[B82] Solarz K. Szilman P. Szilman E. (1999). “Allergenic mites associated with bird nests in Poland (Astigmata: Pyroglyphidae, Acaridae, Glycyphagidae),” in *Proceedings of the 3rd Symposium of the European Association of Acarologists: Ecology and Evolution of the Acari, Series Entomologica, Vol. 55, 1–5 July 1996, Amsterdam* eds Bruin J. van der Geest L. P. S. Sabelis M. W. (Boston, MA: Kluwer Academic Publishers) 651–656. 10.1007/978-94-017-1343-6_56

[B83] Spieksma F. T. M. (1997). Domestic mites from an acarologic perspective. *Allergy* 52 360–368. 10.1111/j.1398-9995.1997.tb01012.x 9188914

[B84] Stepien Z. Rodriguez J. G. (1973). Collecting large quantities of acarid mites. *Ann. Entomol. Soc. Am.* 66 478–480. 10.1093/aesa/66.2.478

[B85] Sun E.-T. Li C.-P. Nie L.-W. Jiang Y.-X. (2014). The complete mitochondrial genome of the brown leg mite, *Aleuroglyphus ovatus* (Acari: Sarcoptiformes): evaluation of largest non-coding region and unique tRNAs. *Exp. Appl. Acarol.* 64 141–157. 10.1007/s10493-014-9816-9 24777358

[B86] Tamura K. Dudley J. Nei M. Kumar S. (2007). MEGA4: molecular evolutionary genetics analysis (MEGA) software version 4.0. *Mol. Biol. Evol.* 24 1596–1599. 10.1093/molbev/msm092 17488738

[B87] Van Asselt L. (1999). Interactions between domestic mites and fungi. *Indoor Built Environ.* 8 216–220. 10.1159/000024644

[B88] van Borm S. Buschinger A. Boomsma J. J. Billen J. (2002). *Tetraponera* ants have gut symbionts related to nitrogen-fixing root-nodule bacteria. *Proc. Biol. Sci.* 269 2023–2027. 10.1098/rspb.2002.2101 12396501 PMC1691126

[B89] Vandekerckhove T. T. M. Watteyne S. Willems A. Swings J. G. Mertens J. Gillis M. (1999). Phylogenetic analysis of the 16S rDNA of the cytoplasmic bacterium *Wolbachia* from the novel host *Folsomia candida* (Hexapoda, Collembola) and its implications for wolbachial taxonomy. *FEMS Microbiol. Lett.* 180 279–286. 10.1111/j.1574-6968.1999.tb08807.x 10556723

[B90] Wang Q. Garrity G. M. Tiedje J. M. Cole J. R. (2007). Naive Bayesian classifier for rapid assignment of rRNA sequences into the new bacterial taxonomy. *Appl. Environ. Microbiol.* 73 5261–5267. 10.1128/AEM.00062-07 17586664 PMC1950982

[B91] Webster L. M. I. Thomas R. H. McCormack G. P. (2004). Molecular systematics of *Acarus siro* s. lat., a complex of stored food pests. *Mol. Phylogenet. Evol.* 32 817–822. 10.1016/j.ympev.2004.04.005 15288058

[B92] Yang B. Cai J. Cheng X. (2011). Identification of astigmatid mites using ITS2 and COI regions. *Parasitol. Res.* 108 497–503. 10.1007/s00436-010-2153-y 21072538

[B93] Zakhvatkin A. A. (1959). *A Translation of Fauna of U.S.S.R. Arachnoidea Tyroglyphoidea (Acari)* Vol. VI, No. 1 eds trans. Ratcliffe A. Hughes A. M. (Washington, DC: The American Institute of Biological Sciences).

[B94] Zindel R. Ofek M. Minz D. Palevsky E. Zchori-Fein E. Aebi A. (2013). The role of the bacterial community in the nutritional ecology of the bulb mite *Rhizoglyphus robini* (Acari: Astigmata: Acaridae). *FASEB J.* 27 1488–1497. 10.1096/fj.12-216242 23307835

