# Supplementary material for: Populations of Stored Product Mite Tyrophagus putrescentiae Differ in Their Bacterial Communities
Source: Front Microbiol. 2016 Jul 12;7:1046. doi: 10.3389/fmicb.2016.01046 (PMC4940368; doi:10.3389/fmicb.2016.01046)
Supplement: Supplementary file 1 [file Table_1.PDF]

Supplemental data Table S1. Operational taxonomic units (OTUs) based on 97% similarity from cloned sequences of bacterial 16S rRNA from populations of *Tyrophagus putrescentiae*.

Journal name: Frontiers in Microbiology

Article title: Populations of *Tyrophagus putrescentiae* Differ in Their Bacterial Communities

Authors names: *Tomas Erban, Jaroslav Smrz, Pavel Klimov, Thomas W. Phillips, Marta Nesvorna, Jan Kopecky and Jan Huber*[1+]

[+]Corresponding author: Jan Hubert,Crop Research Institute, Drnovska 507/73, Praha 6-Ruzyne, CZ-16106, Czechia, e-mail: hubert@vurv.cz

Supplemental data Table S1. Operational taxonomic units (OTUs) based on 97% similarity from cloned sequences of bacterial 16S rRNA from populations of *Tyrophagus putrescentiae*. The sequences originate from clones of eubacterial (F24/R1492) primers amplicons. were identified using Ribosomal Database Project RDP (Wang et al., 2007) training set No. 14 (confidence thresholds are in parentheses). The representative sequences were compared to those in GenBank and the names of taxa of the most similar sequences are presented with accession numbers (A.No.). The similarity (%) is in parenthesis; * indicates sequences for which we found high similarity (97-100%) to sequences of *Bartonella*-like[(1)], *Cardinium*[(2)] and *Solitalea*-like[(3)] bacteria identified in *Acarus siro* and *Tyrophagus putrescentiae* (Hubert et al, 2012; Kopecky et al, 2013). The populations of *Tyrophagus putrescentiae* are described in Table. 1.

	RDP identification	GenBank identification		Populations					
$OTUs_{97}$		Species	A. No.	Ham	Kop	Lab	Dog	Phi	Zvo
Otu001	Paenochrobactrum(65)	*Bartonella rattaustraliani* (96*[1])	NR_116175	6	31	70	9	12	0
Otu002	Bacillus(100)	*Bacillus cereus* (100)	LN890173	0	9	50	2	0	0
Otu003	Enhydrobacter(100)	*Moraxella osloensis* (99)	AJ505859	57	0	0	0	0	0
Otu004	Anaplasma(100)	*Wolbachia* (97)	KJ786949	0	0	0	14	39	0
Otu005	Staphylococcus(100)	*Staphylococcus cohnii* (99)	HM582690	0	0	15	0	3	3
Otu006	Sporocytophaga(95)	Bacteroidetes endosymbiont of *Metaseiulus occidentalis* (*Cardinium*)(98*)	AY753169	6	1	12	0	0	0
Otu007	Ornithobacterium(100)	*Blattabacterium* sp. (91*[2])	NR_102837	0	0	0	14	0	0
Otu008	Arcticibacter(100)	*Solitalea canadensis* (85*[3])	NR_074099	0	1	0	4	5	0
Otu009	Kocuria(100)	*Kocuria* sp. (99)	FJ807674	0	0	8	0	0	1
Otu010	Microbacterium(100)	*Microbacterium oxydans* (99)	NR_044931	8	0	0	0	0	0
Otu011	Brenneria(100)	*Erwinia oleae* (94)	NR_108848	0	0	7	0	0	0
Otu012	Pseudomonas(100)	*Pseudomonas putida* (99)	AP013070	0	0	5	0	0	0
Otu013	Sphingomonas(100)	*Sphingomonas leidyi* (99)	NR_025324	0	0	0	0	0	3
Otu014	Afipia(100)	*Afipia birgiae* (99)	NR_025117	0	0	0	0	0	3
Otu015	Brevibacterium(100)	*Brevibacterium oceani* (98)	NR_042458	0	3	0	0	0	0
Otu016	Natronobacillus(67)	*Pseudogracilibacillus auburnensis* (95)	KR153879	0	0	3	0	0	0
Otu017	Bacillus(100)	*Bacillus thermolactis* (99)	NR_115226	0	0	0	0	0	2
Otu018	Staphylococcus(100)	*Staphylococcus lentus* (99)	NR_043418	0	2	0	0	0	0
Otu019	Paenochrobactrum(100)	*Paenochrobactrum glaciei* (99)	NR_112750	0	0	0	0	0	2
Otu020	Sphingobacterium(100)	*Sphingobacterium psychroaquaticum* (96)	NR_108297	0	0	0	0	0	2
Otu021	Pseudomonas(100)	*Pseudomonas oryzihabitans* (99)	NR_117269	0	0	0	0	2	0
Otu022	Massilia(100)	*Massilia timonae* (97)	NR_026014	0	0	0	0	2	0
Otu023	Arcticibacter(100)	*Solitalea canadensis* (84*[3])	NR_074099	0	0	0	0	1	0
Otu024	Lysinibacillus(100)	*Lysinibacillus macroides* (99)	NR_114920	0	0	0	0	0	1
Otu025	Sporosarcina(100)	*Sporosarcina globispora* (97)	NR_029233	0	0	0	0	0	1
Otu026	Alcaligenes(100)	*Alcaligenes faecalis* (99)	NR_113606	0	0	0	0	0	1
Otu027	Pedobacter(100)	*Pedobacter bauzanensis* (93)	NR_117231	0	0	0	0	0	1
Otu028	Virgibacillus(100)	*Virgibacillus* sp. (95)	KJ144820	0	0	1	0	0	0
Otu029	Virgibacillus(100)	*Oceanobacillus massiliensis* (95)	HQ586890	0	0	1	0	0	0
Otu030	Pseudomonas(100)	*Pseudomonas* sp. (99)	EU370417	0	0	0	0	0	1
Otu031	Sphingomonas(100)	*Sphingomonas* sp. (99)	EU337119	0	0	0	0	0	1
Otu032	Ochrobactrum(100)	*Bartonella elizabethae* (97)	AB246801	0	0	0	0	0	1
Otu033	Pseudochrobactrum(100)	*Pseudochrobactrum kiredjianiae* (97)	KT203923	0	0	0	0	0	1
Otu034	Brevundimonas(100)	*Brevundimonas vesicularis* (99)	KF501480	0	0	0	0	0	1
Otu035	Virgibacillus(100)	*Virgibacillus natechei* (96)	NR_132721	0	0	1	0	0	0
Otu036	Carnobacterium(100)	*Carnobacterium divergens* (99)	LC097075	0	0	0	0	0	1
Otu037	Bacillus(100)	*Bacillus fortis* (95)	NR_042905	0	0	0	0	0	1
Otu038	Stenotrophomonas(100)	*Stenotrophomonas rhizophila* (99)	NR_121739	0	0	0	0	1	0
Otu039	Enterobacter(100)	*Enterobacter ludwigii* (96)	NR_042349	0	0	0	0	0	1
Otu040	Acinetobacter(100)	*Acinetobacter radioresistens* (99)	NR_114074	0	0	0	0	1	0
Otu041	Escherichia/Shigella(100)	*Escherichia coli* (99)	LN831047	1	0	0	0	0	0
Otu042	Paenochrobactrum(100)	*Bartonella rattaustraliani* (92)	NR_116175	0	0	1	0	0	0

Supplementary references:

Hubert, J., Kopecky, J., Perotti, M. A., Nesvorna, M., Braig, H. R., Sagova-Mareckova, M., Macovei, L., and Zurek, L. (2012). Detection and identification of species-specific bacteria associated with synanthropic mites. *Microb. Ecol.* 63, 919–928

Kopecky, J., Perotti, M. A., Nesvorna, M., Erban, T., and Hubert, J. (2013). *Cardinium* endosymbionts are widespread in synanthropic mite species (Acari: Astigmata). *J. Invertebr. Pathol.* 112, 20–23.

Wang, Q., Garrity, G. M., Tiedje, J. M., and Cole, J. R. (2007). Naive Bayesian classifier for rapid assignment of rRNA sequences into the new bacterial taxonomy. *Appl. Environ. Microbiol.* 73, 5261–5267.
